# Creating respiratory pathogen-free environments in healthcare and nursing-care settings: a comprehensive review

**DOI:** 10.1007/s11357-024-01379-7

**Published:** 2024-10-11

**Authors:** Attila Nagy, Aladár Czitrovszky, Andrea Lehoczki, Árpád Farkas, Péter Füri, János Osán, Veronika Groma, Szilvia Kugler, Adrienn Micsinai, Alpár Horváth, Zoltán Ungvári, Veronika Müller

**Affiliations:** 1 Department of Applied and Nonlinear Optics, HUN-REN Wigner Research Centre for Physics, Konkoly-Thege Miklós St. 29-33, 1121 Budapest, Hungary; 2 Doctoral College, Health Sciences Program, Semmelweis University, Budapest, Hungary; 3 Institute of Preventive Medicine and Public Health, Semmelweis University, Budapest, Hungary; 4 Environmental Physics Department, HUN-REN Centre for Energy Research, Budapest, Hungary; 5 Eurofins BIOMI Ltd, Gödöllő, Hungary; 6 Department of Pulmonology, Semmelweis University, Budapest, Hungary; 7 Vascular Cognitive Impairment and Neurodegeneration Program, Oklahoma Center for Geroscience and Healthy Brain Aging, Department of Biochemistry & Molecular Biology, University of Oklahoma Health Sciences Center, Oklahoma City, OK 731042 USA; 8 Peggy and Charles Stephenson Cancer Center, Oklahoma City, OK 73104 USA; 9 Department of Health Promotion Sciences, College of Public Health, University of Oklahoma Health Sciences Center, Oklahoma City, OK USA; 10 International Training Program in Geroscience, Doctoral School of Basic and Translational Medicine/Institute of Preventive Medicine and Public Health, Semmelweis University, Budapest, Hungary

**Keywords:** Virus-containing aerosol particles, Exhaled particles, Droplets, Airway deposition, Bioaerosol, Sampling, Air filtration

## Abstract

Hospital- and nursing-care-acquired infections are a growing problem worldwide, especially during epidemics, posing a significant threat to older adults in geriatric settings. Intense research during the COVID-19 pandemic highlighted the prominent role of aerosol transmission of pathogens. Aerosol particles can easily adsorb different airborne pathogens, carrying them for a long time. Understanding the dynamics of airborne pathogen transmission is essential for controlling the spread of many well-known pathogens, like the influenza virus, and emerging ones like SARS-CoV-2. Particles smaller than 50 to 100 µm remain airborne and significantly contribute to pathogen transmission. This review explores the journey of pathogen-carrying particles from formation in the airways, through airborne travel, to deposition in the lungs. The physicochemical properties of emitted particles depend on health status and emission modes, such as breathing, speaking, singing, coughing, sneezing, playing wind instruments, and medical interventions. After emission, sedimentation and evaporation primarily determine particle fate. Lung deposition of inhaled aerosol particles can be studied through in vivo, in vitro, or in silico methods. We discuss several numerical lung models, such as the Human Respiratory Tract Model, the LUng Dose Evaluation Program software (LUDEP), the Stochastic Lung Model, and the Computational Fluid Dynamics (CFD) techniques, and real-time or post-evaluation methods for detecting and characterizing these particles. Various air purification methods, particularly filtration, are reviewed for their effectiveness in healthcare settings. In the discussion, we analyze how this knowledge can help create environments with reduced PM2.5 and pathogen levels, enhancing safety in healthcare and nursing-care settings. This is particularly crucial for protecting older adults, who are more vulnerable to infections due to weaker immune systems and the higher prevalence of chronic conditions. By implementing effective airborne pathogen control measures, we can significantly improve health outcomes in geriatric settings.

## Introduction

The COVID-19 pandemic has resulted in a significant global health crisis, with substantial morbidity and mortality [1–4]. As of mid-2024, there have been over 600 million confirmed cases and more than 6 million deaths worldwide [1]. The pandemic has highlighted the importance of understanding aerosol transmission of respiratory pathogens and its implications for public health. Studies have shown that airborne particles are a major route of transmission, particularly in enclosed spaces with poor ventilation [5–7]. This awareness has spurred extensive research into the behavior of airborne particles in healthcare settings and the development of strategies to mitigate the spread of airborne pathogens [6, 8–11].

SARS-CoV-2, the virus responsible for COVID-19, can be transmitted via aerosols generated through normal respiratory activities, such as breathing, speaking, coughing, and sneezing [5–7]. Patient-derived aerosols are also primary vectors for many other respiratory pathogens like influenza, respiratory syncytial virus (RSV), and *Mycobacterium tuberculosis* [12–17]. These aerosols, especially those smaller than 50 to 100 µm, can remain airborne for extended periods, facilitating the spread of these viruses and bacteria over distances greater than initially anticipated.

Activities like speaking, singing, and coughing generate aerosols that can carry infectious particles, making infection control within healthcare settings a critical concern. Understanding the dynamics of these aerosols—from their formation in the respiratory system to their deposition in the lungs—is essential for developing effective strategies to mitigate airborne transmission.

In addition to patient-derived aerosols, air pollution-related particulate matter (PM) can exacerbate respiratory infections. Aerosol PM is a significant component of air pollution with notable toxicological effects due to the presence of substances like metals, nitrates, and sulfates on particle surfaces. These particles can irritate the respiratory tract, leading to inflammation and reduced immune response, which makes the body more susceptible to infections. Fine particles (PM2.5) can penetrate the circulatory system, posing serious health risks, such as cardiovascular and heart diseases, respiratory infections, chronic lung diseases, cancers, and preterm births [18–20]. Long-term exposure to PM2.5 has been linked to higher mortality rates from COVID-19, suggesting that air pollution can significantly impact the severity of respiratory infections [21]. Furthermore, air pollution-related PM can carry pathogens, potentially increasing the risk of infection. Fine particles have large surface-to-volume ratios and can adsorb airborne viruses to form virus-laden aerosols, mediating the spread of various viral infections [22–29]. The COVID-19 pandemic has amplified concerns regarding PM and its role in airborne virus transmission [30]. Severe acute respiratory syndrome coronavirus 2 (SARS-CoV-2) viral RNA was identified on the surface of PM from public locations [30]. Therefore, addressing both patient-derived aerosols and air pollution-related PM is crucial for comprehensive infection control.

Older adults have been disproportionately affected by the COVID-19 pandemic [2, 31]. The majority of COVID-19 victims were geriatric patients, underscoring the vulnerability of this population [2, 31]. Older adults, particularly those in geriatric settings, are also highly susceptible to other respiratory infections, including influenza [32–34]. Several physiological and environmental factors contribute to this increased risk. The immune system’s efficiency declines with age, making it harder for older individuals to combat infections. This immunosenescence leads to higher susceptibility to respiratory pathogens [31, 32, 35–37]. Additionally, chronic conditions such as diabetes, heart disease, and chronic obstructive pulmonary disease (COPD) are more prevalent among older adults, further compromising their ability to recover from infections [2, 3]. Geriatric care facilities, where residents live in close quarters and often have compromised health, are particularly at risk for rapid spread of infections. Protecting older adults from airborne pathogens in these settings is paramount to reducing morbidity and mortality. Older adults are frequently hospitalized for various reasons, including chronic diseases as well as for post-operative rehabilitation following surgeries. During these hospital stays, they are at an increased risk of encountering respiratory pathogens due to their weakened immune systems and the high prevalence of infectious agents in hospital environments [38]. Long-term care settings, where older adults receive ongoing medical attention, further increase their exposure to potential infections. Additionally, the need for frequent medical interventions and procedures in this demographic can lead to increased aerosol generation, amplifying the risk of airborne transmission.

In hospital settings, older adults are often in close proximity to other patients, healthcare workers, and visitors, all of whom can be sources of respiratory pathogens. The combination of prolonged exposure, frequent medical interactions, and compromised health makes infection control measures in these environments crucial. Effective infection control in geriatric settings requires a comprehensive understanding of the dynamics of aerosol transmission. This includes recognizing the various activities that generate aerosols and understanding how these particles behave in the air. Moreover, given the heightened vulnerability of older adults, special attention must be given to air quality management, including the use of advanced air purification systems and rigorous monitoring of airborne pathogens. By addressing these factors, we can significantly improve health outcomes and reduce the risk of airborne diseases in geriatric and hospital settings.

This review aims to synthesize current literature on the dynamics of respiratory particle transmission, focusing on patient-derived aerosols as the primary vectors for pathogens such as influenza and SARS-CoV-2. By exploring the formation, transport, and deposition of these particles, we seek to provide a detailed understanding of the mechanisms driving airborne transmission. The review also covers the role of air pollution-related PM in exacerbating respiratory conditions and its potential interaction with pathogens. This dual focus highlights the need for comprehensive air quality management strategies in healthcare and geriatric settings. Furthermore, we discuss various detection methods for airborne pathogens, the spread of these particles in different environments, and the effectiveness of air purification techniques. By integrating these insights, the review provides practical recommendations for creating safer environments in healthcare and nursing-care settings, with a particular emphasis on protecting older adults.

## Airway origin of virus-containing aerosol particles

Until the early twentieth century, respiratory diseases were thought to be spread by direct contact or by larger droplets. In the middle of the twentieth century, investigators showed that particles leave the respiratory system during coughing and sneezing, measured their size distribution, and investigated the atomization process [39–44]. Due to the limitation of their measurement equipment, they found that these particles fell to the floor near the source because of their size distribution. Subsequently, from the 1980s, it became evident that a significant number of submicrometer-sized particles leave the respiratory system remaining in the air for a longer time [45–47] and may therefore play an important role in the transmission of infections.

Since then, it has become more widely accepted that the airborne route is the primary way of transmission of many well-known pathogens, like the influenza virus [48] and SARS-CoV-2 [49, 50]. The dynamics of airborne virus transmission are not fully understood [51]. The main challenges are related to the complicated fluid and flow characteristics involved in the transport of virus-carrying droplets and aerosol particles [52]. Source dynamics (e.g., exhale velocity and temperature, droplet sizes, viral load), ambient conditions (e.g., laminar and turbulent flows, temperature, and humidity), and virus dynamics (e.g., virus viability and infection rate) have to be taken into consideration.

### Mechanisms of particle generation

Human airways are not only a region for particle deposition but also a site of origin for particles that can leave the airways through various respiratory activities. It has been shown that not only sneezing and coughing but also laughing, speaking, playing wind instruments, singing, or even normal breathing can produce aerosol particles [53–57]. These particles form in different regions of the respiratory tract by different mechanisms [58–61]. Particles are also generated during medical interventions (e.g., bronchoscopy, spirometry, dentistry). After particle generation, they can float in the air for a long time [62, 63], ready to be inhaled again by someone in close proximity. The range of time respiratory particles can spend in the air spans from minutes (for 5–10 µm size particles) to hours or even days (for submicron particles). According to the findings of studies measuring the sizes and concentrations of the particles generated during medical processes (e.g., [64–68]), the amount of submicron particles is substantial. Even the World Health Organization (WHO) needed some time to change its terminology and clarify the definitions for droplets and aerosol particles, finally leading to the acceptance of the airborne transmission of respiratory viruses [69, 70]. This change in thinking was governed by the scientific community’s findings on the airborne lifetime of particles with different sizes [61, 71, 72]. The critical size below which a particle remains airborne for a longer period and plays an important role in pathogen transmission is around 50–100 µm, but it was initially thought to be 5 µm. In our terminology, a respiratory particle refers to a droplet above this critical size of 100 µm, while a particle below this size is classified as an aerosol particle.

### Factors influencing particle properties

Particles emitted from the airways were extensively studied in the past. However, the research area became a hot topic after the outbreaks of Severe Acute Respiratory Syndrome (SARS) in 2002 and Middle East Respiratory Syndrome (MERS) in 2012, but especially after the onset of the COVID-19 pandemic in 2019–2020. It has been demonstrated that the physicochemical properties of the emitted particles are highly dependent, among others, on the health status, mode of emission (e.g., breathing, coughing, speaking [60]), but also on the intensity of the activity (normal talk versus shouting, quiet breathing versus breathing during heavy physical activity [73, 74]). In addition, concentration, size distribution, content, and other characteristics of the emitted particles may exhibit important inter-individual variability. For instance, healthy subjects emit a considerable number of particles, but infected individuals may expel more particles with different size distributions [75].

### Regional origin and characteristics of particles

Virus-containing particles can originate from any region of the airways, with their number, size, and virus content at the moment of emission depending on the specific site within the respiratory tract [58, 76]. Different mechanisms predominate in different anatomical regions [59–61].

#### Upper airways

The main mechanism for generating droplets in the upper airways is the breakup of viscoelastic filaments. This means that the viscoelastic fluid in this airway region can undergo stretching, leading to the potential fragmentation of the filaments as exhaled airflow passes by. For instance, the saliva filaments that form during mouth opening can bend and break during exhalation, speaking, or singing. As this phenomenon happens at the level of lips, it is possible to visualize it directly by high-speed imaging [77]. Though this process is rather complex and multifaceted, it is hypothesized that mechanical properties of mucus (especially elasticity) and other dynamic conditions influence the flattening of expelled saliva into a sheet that can destabilize into ligaments and droplets with the details of a breakup being caused by an interplay of inertia and elasticity [78].

#### Vocal cords

Vocalization generates droplets at the level of the voice box through the vibration of the vocal folds, creating liquid filaments that break into droplets, which are then emitted (76).

#### Bronchioli

In the deeper regions of the lungs, particularly the bronchioli, particles are formed primarily through the bursting of mucus filaments. Elasto-capillary instability of mucosal films is considered to play a key role in this process. As air velocity is low in this part of the airways, the breakup is caused by the expansion of the airways during inhalation (small airway reopening) rather than the inertial effect of the airflow [79]. Some of the particles that are formed by this process during inhalation will leave the airways during the exhalation phase.

The site of origin significantly influences the size of the generated particles. Generally, the deeper the location of droplet formation in the airways, the smaller the particle size. Consequently, the largest particles and droplets are released from the mouth (10–100 µm and even larger). These particles are larger than those originating from the laryngeal region (mainly between 1 and 10 µm), though the particle formation mechanism is the same in these two regions. As air velocity plays a role in these cases, the different cross-sectional areas characterized by different air velocities may explain this. The length scale of the mouth opening is at least one order of magnitude higher than the length scale of the vocal fold’s distance when the filaments and droplets are formed. Particles from the bronchioli are the smallest, typically in the range of 0.7–1 µm [79]. The number and location of these modes are influenced by multiple factors, including the site of particle formation [58]. Understanding the mechanisms of particle and droplet generation and the factors influencing their properties is crucial for developing strategies to mitigate airborne transmission of respiratory pathogens. This knowledge is particularly important for protecting vulnerable populations, such as older adults in healthcare and geriatric settings, from infections.

## Fate of exhaled particles and droplets in the air

### Environmental influences on particle pathway

When particles leave the respiratory system, they are immediately subjected to various environmental influences that affect their trajectory and fate in the air. The two primary processes that determine the behavior of aerosol particles and droplets are sedimentation (gravitational settling) and evaporation. These processes are influenced by several physical and chemical factors, including temperature, relative humidity, initial droplet radius, initial solute volume fraction, and the height from which the droplet is emitted [80].

#### Sedimentation

Sedimentation is primarily governed by the diameter of the particles. Larger droplets, typically those with diameters greater than 50–100 µm, settle quickly due to gravitational forces, reducing the likelihood of inhalation beyond a few meters from the source. According to the work of Wells, the air resistance becomes negligible for droplets larger than 100 µm [39]. These big droplets exhibit a mainly ballistic type of behavior. In contrast, smaller particles with diameters of a few micrometers can remain airborne for extended periods, sometimes lasting hours [62, 63] (see Table 1).

**Table 1:** Settling of water particles and droplets in saturated air [39]

Droplet diameter (mm)	Time in seconds of falling 2 m
> 1 mm	0.6
0.1	6.0
0.01	600 (10 min)
0.001	60, 000 (16.6 h)

#### Evaporation

Evaporation significantly affects the size and behavior of respiratory droplets and particles. Droplets and aerosol particles emitted from the respiratory tract consist of water and non-volatile substances like proteins, enzymes, and electrolytes. These particles can also carry pathogens such as Mycobacterium tuberculosis, Influenza virus, or SARS-CoV-2. In typical room conditions, evaporation reduces the diameter of these particles to about 20–50% of their original size, depending on the non-volatile content and ambient relative humidity [81].

### Sedimentation vs. evaporation

Many studies presented numerical models that tried to describe the interplay of sedimentation and evaporation of the particles emitted from the respiratory tract (e.g., [52, 80, 82, 83]). One of the earliest and most cited works is the study of [39]. Although the Wells model neglects some important physical–chemical aspects of evaporation and sedimentation, scientists generally agree that the initial size of the particles is important, as described in the following two subsections (“Comparison of settling times and evaporation rates” and “Impact of non-volatile content on particle behavior”) [80].

#### Comparison of settling times and evaporation rates

It is crucial to compare the settling times of particles with their evaporation rates to understand their behavior in the air. Emitted from a specific height in still-standing saturated air (the relative humidity RH = 1), the particle’s diameter is the most important parameter that determines the sedimentation time [39]. For instance, Wells [39] showed that large droplets (> 100 µm) have negligible air resistance and exhibit ballistic behavior, meaning they fall quickly to the ground. Smaller particles reach their terminal velocity rapidly and can stay airborne for much longer periods (see Table 1). Table 2 shows how long water droplets can remain in unsaturated air at 18 °C before evaporating completely [39]. Comparing the values in Tables 1 and 2, the evaporation of particles smaller than 0.1 mm is faster than their sedimentation from 2 m height. Without non-volatile content, the small particles would not reach the ground. In reality, the aerosol particles emitted from the respiratory tract always have some non-volatile content.

**Table 2:** The duration water droplets can stay in unsaturated air at 18 °C before they completely evaporate [39]

Droplet diameter (mm)	Evaporation time in seconds
2	660
1	165
0.5	41
0.2	6.6
0.1	1.7
0.05	0.4

It is quite difficult to describe the complex physical–chemical effects that drive sedimentation and evaporation, so most studies simplify or neglect some of these processes. For example, in the 140 nm–120 μm particle diameter range, evaporation can be described in the stagnant-flow approximation and considered to be diffusion-limited [62]. Using these assumptions, Netz presented equations for the droplet evaporation rate, the time-dependent particle size, and the sedimentation time, including evaporation cooling and solute osmotic-pressure effects [62]. According to this model, the sedimentation time of particles with different sizes for RH = 1 and RH = 0.5 with a 1% non-volatile fraction in the droplet or particle is listed in Table 3.

**Table 3:** Representative sedimentation times for RH = 1 (*t*_sed_) and RH = 0.5 with 1% non-volatile content (*t*_sed_^sol^) from 2 m [62]

Initial diameter (µm)	1	2.5	5	10	20	30	40	55
*t*_sed_ RH = 1	5 h	45 min	11 min	170 s	43 s	19 s	11 s	5.6 s
*t*_sed_^sol^ RH = 0.5	64 h	10 h	154 min	38 min	9 min	231 s	99.6 s	7.6 s

#### Impact of non-volatile content on particle behavior

The presence of non-volatile content in respiratory droplets affects both sedimentation and evaporation. Particles with significant non-volatile content do not evaporate completely and can remain airborne for longer durations. Studies have modeled these interactions, showing that in the 140 nm–120 µm diameter range, evaporation can be diffusion-limited [62]. These models provide equations for droplet evaporation rates, time-dependent particle sizes, and sedimentation times, considering evaporation cooling and solute osmotic-pressure effects.

Data from such models (see Table 3) indicate that for particles with diameters smaller than 30–40 µm and containing 1% non-volatile content, settling times can range from minutes to hours, especially at lower relative humidity levels (RH = 0.5). Particles with non-volatile content reach an equilibrium size during evaporation and thus remain in the air for a longer time. These prolonged airborne periods increase the likelihood of inhalation and pathogen transmission, highlighting the significance of these small aerosol particles in disease spread [80].

### On the viability of pathogens

Microbial survival is even possible in the stratosphere in extreme conditions, including tolerance to intense UV radiation, low pressure, lack of water and nutrients, and freezing temperatures [84]. Fortunately, most pathogens, like viruses, do not survive even in less extreme conditions. Van Doremalen et al. evaluated the stability of SARS-CoV-2 and SARS-CoV-1 in aerosols and on various surfaces [85]. SARS-CoV-2 remained viable in aerosols throughout the experiment (3 h), and the virus half-life was 1.1 to 1.2 h. The SARS-CoV-2 virus can survive a pretty long time on multiple surfaces. According to the data of Gidari et al. [86], infectivity was maintained on plastic and glass for 120 h and on stainless steel for 72 h. The virus half-life was 5.3 h on plastic, 4.4 h on stainless steel, and 4.2 h on glass surfaces. In all cases, titer decay was > 99% after drop drying.

### On the role of fomites in pathogen transmission

Early in the COVID-19 pandemic, there was a period when some infectious disease clinicians blamed physical contact with fomites over airborne transmission. After a while, it became proven and widely accepted that airborne transmission is the primary route. However, the role of fomites should not be abandoned. Disinfection of surfaces and frequent hand washing is effective against infection through direct contact with fomites. However, there was an extensive discussion on the role of fomites and airborne particles in virus transmission and infection. Indeed, the two have a strong connection, as viable virus-laden droplets can settle on objects quickly and be re-suspended. The exhaled droplets settle on surrounding surfaces, clothes, etc., within minutes, where the viruses can survive for hours. This time is sufficient for the viruses to survive until they are reintroduced into the air by resuspension. For instance, our previous studies demonstrated that medical staff activities increased the concentration of airborne particles originating from the re-suspension of the particles from different surfaces [87, 88].

In summary, understanding the environmental influences on exhaled particles and droplets and the viability of the carried pathogens is essential for comprehending their role in airborne transmission. The dynamics of sedimentation and evaporation, along with factors like temperature and relative humidity, play critical roles in determining how long particles remain airborne and their potential to transmit diseases. This knowledge is crucial for developing effective infection control strategies, particularly in healthcare and geriatric settings, where the risk of airborne pathogen transmission is high.

## Airway deposition of inhaled particles

### Dynamics of particle deposition

#### Interaction with airway surfaces

The respiratory tract serves as a portal of entry for inhaled materials, with particles depositing on the extensive airway surface and interacting with it [89]. Upon inhalation, particles can deposit along various regions of the airways, including the nasal passages, trachea, bronchi, and deeper alveolar regions. The interaction of these particles with airway surfaces can lead to a range of health effects, depending on the nature of the particles and the site of deposition.

#### Health effects of deposited particles

In addition to transmitting viruses, bacteria, and fungal spores, inhaled particles can be detrimental, as they may include air pollutants, toxic particles, and pollens, all of which can induce various adverse health effects [90]. These health effects can range from mild respiratory irritation to severe diseases such as chronic obstructive pulmonary disease (COPD) and asthma. Additionally, therapeutic aerosols administered via the airways are crucial for managing several airway diseases, and there is increasing research to facilitate drug delivery to other organs via the lungs and systemic circulation [91–93].

### Methods to determine deposition

The lung is a complex system of bifurcating airways. For instance, assuming no termination before generation 25, the number of airway ducts is around 2^25^ = 33.5 million in the 26th airway generation (the trachea corresponds to generation 1). As we descend from the trachea to the terminal bronchus (which is between airway generations 12–21), the number of airways increases while their size diminishes significantly. The diameter and the length of the first bronchial airway (main bronchus) are 12 mm and 38 mm, respectively. However, the 11th alveolar duct is quite small; it has a diameter of 0.28 mm and a length of only 0.17 mm [94]. The small size and large number of overlapping airways make it difficult to measure the deposited amount of the inhaled particles in the acinar section of the lung by a gamma camera or by single photon emission computer tomography (SPECT).

The deposition distribution of inhaled aerosol particles in the human airways can be determined through three primary methods: in vivo measurements, in vitro studies, and simulations.

In vivo methods involve direct measurements within living organisms, typically using imaging techniques like gamma cameras or single photon emission computed tomography (SPECT) to visualize and quantify particle deposition within the respiratory tract.

In vitro studies utilize lung models or airway replicas to simulate and measure particle deposition. These methods provide controlled environments to study specific variables affecting particle behavior.

Simulation tools, such as numerical lung models or Computational Fluid Dynamics (CFD) techniques, offer a unique way to investigate the airway deposition of aerosol medicines, inhaled radon progeny, or any other particles found in the air of homes, workplaces, hospitals, or nursing homes. Simulations are repeatable, reproducible, and cost-effective, and they have the advantage that the input data (e.g., airway geometry and breathing mode) can be selected optionally. In the past, the primary constraint of the simulations was the available computational power. Over the last three decades, the number of operations executed per second has increased to such an extent that today’s computational capabilities allow for considerably more demanding simulations compared to those feasible in the 1990s.

### Numerical lung models

Numerical lung models have quite a long history. Findeisen was the first to describe the deposition distribution of inhaled particles [95]. Landahl increased the number of bronchial and acinar airways, redefined the deposition formulas, and developed a method to simulate the effect of inhaled volume on the deposition distribution [96]. The next step was to integrate the morphological model of Weibel into Landahl’s lung model [97]. One year later, the first lung model of the International Commission on Radiological Protection (ICRP) was presented. In 1994, the ICRP presented a new lung model, the Human Respiratory Tract Model (HRTM) [94], while the National Council on Radiation Protection and Measurements (NCRP) presented another lung model (LUDEP) similar to the ICRP HRTM in 1997. The HRTM and the LUDEP are regional compartment models, meaning that the deposition of inhaled particles can be calculated only in some larger regions of the respiratory tract. For example, the HRTM can only calculate the extrathoracic, the bronchial, the bronchiolar, and the acinar deposition. However, the deposition distribution of the particles is usually strongly inhomogeneous in the lungs. To determine the deposited amount of particles in each compartment, the HRTM model uses empirical deposition formulas, which were determined from experimental data on lung casts and on aerosol airway deposition experiments done with volunteers.

To better describe the complicated structure of the lung, [98] presented the stochastic version of the ICRP66 model (LUDUC).

Furthermore, [99] presented the Multiple Path Particle Deposition Model, which, unlike the HRTM and the NCRP lung model, had the capability to describe the asymmetric branching structure of the bronchial airways in the lung. The most detailed and flexible numerical lung model in the literature is the Stochastic Lung Model (SLM) created by Koblinger and Hofmann between 1985 and 1990 [100–103]. This model uses the Lovelace database [104] to describe the bronchial airways’ geometry (length, diameter, branching, and gravitational angle). With the simulation of the main particle deposition mechanisms, impaction, sedimentation, and diffusion in large numbers (usually ten to hundred thousand) of individual particle pathways, this model can describe the asymmetric branching of the airways. Due to the developments made over the past three decades, the SLM has also gained notable flexibility. The current version of the SLM is able to describe the deposition distribution of the inhaled particles not only for healthy adults but for diseased (asthmatic, emphysematic) lung as well. By downscaling the airways, the model is capable to perform deposition distribution simulations for children too. This model was successfully used to determine the deposition distribution of aerosol medicines [105], ultrafine urban dust [106] as well as respiratory droplets/droplet nuclei [87, 107, 108].

CFD models can calculate the deposition distribution of the inhaled particles with high spatial resolution [109], but these simulations often require high computational power, and precise specification of the airway geometry is essential. Due to a lack of input data (specifically airway geometry) and the need to expedite CFD simulations, this approach frequently entails significant simplification of the acinar region of the lung during the airway deposition simulations.

## Measurement techniques for exhaled respiratory particles

With advances in measurement methods and technology, our understanding of the particles leaving the respiratory system has also changed significantly. Initially, it was thought that particles larger than one micron were mainly produced and released from the respiratory system, which was supported by early observations using glass slides or filters and microscopes to determine the size distributions [41–44]. High-speed photography was also utilized to reveal the atomization process at the mouth and nose [15]. The above techniques have the limitation that only particles larger than one or ten microns can be observed. Later, since the 1980s, optical particle counters (OPC) and aerodynamic particle sizers (APS), based on light scattering methods, have been used to study the emitted particles with increasing sensitivity and time resolution [45–47]. It was observed that contrary to previous results, the majority of the expiratory particles are in the submicron size range. Since a relatively small number of particles are exhaled, measurements must be carried out in a clean room environment, which has been achieved in different ways by different research teams. Johnson and Morawska’s group built a laminar-flow tube system to fit the subjects’ heads and measured exhaled particles in a filtered clean air stream with an APS [53, 58, 110]. Gregson’s group also used APS for the measurements, which were carried out in an ultra-clean hospital operating theater [60]. They compared the particles exhaled during singing to those emitted during speech and normal breathing. The APS measures particles with an aerodynamic diameter between 0.5 and 20 µm. Holmgren’s group measured particles below 0.5 µm using a scanning mobility particles sizer (SMPS) alongside an OPC, which measure exhaled particles from 10 nm and 0.4 µm, respectively [59]. Due to the longer measurement time of SMPS, the particles were collected in a temperature-controlled container. Low particle numbers resulted in noisy data [59] or concentrations just below the detection limit of the SMPS [47].

The results suggest that particles exiting the respiratory system have a multimodal size distribution, with different modes being generated in different lung regions [53, 58, 76, 79]. Although the majority of the particles are in the submicrometer range, particles larger than a couple of microns, mostly produced during coughing or sneezing, represent a significant mass or volume [111], which becomes particularly important in pathogen transmission.

However, when studying their pathogen transmission properties, size distribution and concentration are not the only parameters that describe the particles. The expiration air jets were studied by a high-speed camera using the interferometric Mie imaging and particle imaging velocimetry (PIV) techniques [112]. The applied techniques allowed the analysis of particles larger than 1 µm, and besides the size distribution, the flow field was also determined in close proximity to the mouth. Other authors also used the PIV and the Schlieren optical techniques to study the transport characteristics of human cough-generated particles [113–115]. Besides initial velocities, the opening angles of the expelled air jets during coughing or speaking were determined in these experiments. The applied technique also allowed the observation of the effects of different masks [114]. The results show that wearing a surgical or N95 mask reduces the risk of infection by blocking the path of particles emitted during coughing (N95 mask) or redirecting them in a less harmful direction (surgical mask).

Using a laser diffraction measurement system, Han’s group showed that a single sneeze could generate up to several tens of thousands of droplets and particles with unimodal (~ 100 µm) and bimodal (~ 100 µm and ~ 400–600 µm) volume size distributions and with velocities upwards of 20 m/s [116]. In contrast, APS measurements showed that coughing generates 10–100 times fewer particles than sneezing, with velocities of ~ 10 m/s, but even during talking, approximately 50 particles per second are emitted from the nose and mouth [73].

At this point, it should be noted that while size distribution measurements taken directly in front of the mouth or nose provide information to estimate the pathogen content of particles, measurements taken further away, where the size is already reduced by evaporation, provide important data to determine the airborne lifetime and dispersion of the particles.

## Sampling methods for indoor aerosol and pathogens

Concerning their potential risks to humans, the concentration and size distribution of aerosol particles carrying pathogens are the key factors that determine the time they spend in the air and their deposition in the human respiratory system. Thus, appropriate methodologies need to be available to evaluate environmental samples with sufficient accuracy. As physical and biological efficiency is required, and clear, standardized sampling methods and associated requirements are not yet available, much work remains to be done in this field [117]. However, the COVID-19 pandemic brought significant attention and advancement opportunities in this field. The most important expectations regarding measurement methods are as follows: (i) to detect the presence of aerosolized viruses and/or bacterial and/or fungal aerosols, (ii) to collect them with adequate efficiency, (iii) to maintain their viability, (iv) to be capable for on-the-spot detection; (v) to have an appropriate (typically high) time resolution. Up to now, the biggest shortcomings of sampling devices are their low collection efficiency for ultrafine particles containing infectious viruses or bacteria [118–120] and the deactivation of pathogens during sampling. In general, it can be stated that the same sampling can be applied regardless of the pathogens studied [121].

### Air sampling methodologies

Several research groups presented a broad summary of the aerosol sampling equipment applied in indoor environments, discussing their advantages and disadvantages [117, 121–125]. Impactors accelerate particles of incoming air stream using small nozzles (slits or holes), and due to their inertia, the particles impact the surface of the collection media within a definite size fraction. Then, the collection media are subjected to subsequent analysis, e.g., virus isolation [122]. In the case of cyclones, similarly to impactors, ambient air is drawn using a vacuum pump, but particles larger than a characteristic diameter impact onto the collection wall due to the centrifugal force. The main advantage of these instruments is that they can collect infectious particles in different size ranges, although better efficiency was found for larger particles (90% versus 40% for PM_10_ and PM_2.5_, respectively). Moreover, due to the nature of the particle collection method (e.g., high air speed inside the sampler), the deactivation of viruses can happen [126]. Modifying conventional samplers shows improvement in small-size particle collection efficiency [127] and mobility [128].

The operating principle of liquid-based impingers is that the aerosol particles are transitioned through a flask containing a liquid by accelerating them through a narrow orifice. Thanks to the fact that these sampling devices maintain the viability of viruses and there is no need to extract viruses from the collection media, this measurement method is the most commonly used. However, the collection efficiency of small particles (< 500 nm) still needs improvement, for which some efforts have been made [117, 129, 130].

Due to their low cost and easy usage, filter packs are also widely used for sampling infectious airborne particles. It was found that filtering is efficient in a wide size range (from 20 nm up to 10 µm), and it can be operated even in a selected size fraction by using a pre-separator, but the viability of bacteria and viruses is not guaranteed. Many different filter types (composition, pore size, and thickness) have been used to sample bioaerosols. Although gelatin filters have the great advantage that the viral infectivity is not affected significantly by the sampling process, it still needs careful handling as it was found to be very sensitive to sampling conditions (temperature and relative humidity) [131]. Polytetrafluoroethylene (PTFE) and cellulose filters are also widely used as alternatives.

An electrostatic precipitator (ESP) is a size-dependent particle collector wherein an air sample is overflowed through a high-voltage corona, where the particles are charged and then precipitated onto a grounded rotating disc. The greatest limitation of this equipment is that the ozone formation inside it deactivates viruses [132].

Water-based condensation particle counters are efficient for even very small (few nanometers) aerosol particles as they first enlarge the particles through condensation of water vapor. Although they maintain the viability of viruses, the large size and the complexity of operation make their widespread application difficult.

### Detection of pathogens

Nucleic acid-based molecular biological diagnostical methods are considered to be the gold standard in detecting SARS-CoV-2. The quantitative reverse transcriptase polymerase chain reaction (qRT-PCR) has proven its strength, such as high specificity and accuracy [133]. The first methods were designed based on a few viral genomic data [134, 135]; however, these were available within five weeks following the reporting of a severe acute respiratory disease in the last months of 2019 in Wuhan, China.

Conventional nucleic acid tests were under tremendous pressure to provide massive amounts of results at the peaks of the pandemic due to their reliance on specialized laboratories. Thus more decentralized, user-friendly, and low-cost methods emerged, such as reverse transcriptase followed by isothermal amplification reaction (loop-mediated isothermal amplification (LAMP), recombinase polymerase amplification (RPA)) [136]; CRISPR – based detection (SHERLOCK, DETECTR, CASdetec) [137]; as well as immunological assays like serological antibody testing and antigen testing [138].

For detecting SARS-CoV-2 viruses from environmental matrices, like surfaces or aerosols, qRT-PCR and reverse transcription digital droplet PCR (RT-ddPCR) emerged as the generally used method [139–142]. Nucleic-based amplification techniques still offer unbeatable accuracy with a limit of detection of 2–150 copies/µl at their best [138].

### Sample collection methods and results

Sampling methods applied for the detection of various pathogens in indoor environments in the past ten years are summarized in Table 4, focusing on hospitals and nursing homes. As a result of the COVID-19 pandemic, there has been a significant increase in the number of publications focusing on the sampling and detection of pathogens. In the case of the SARS-CoV-2 virus, it can also be observed that after the initial negative test results, the detection of infected particles improved due to the more sophisticated sampling procedures and selections of operation parameters (such as flow rate). Table 4 summarizes exclusively studies for which at least one positive result was obtained during the series of measurements. However, the standardization of measurement and detection procedures is still lacking [143]. It is important to highlight that information about a wide size range of aerosol particles (from nanoparticles up to large particles of several micrometers) and the maintenance of viability are the two most important issues that should be solved to move forward.

**Table 4:** Air sample collection methods and results in hospital and healthcare facility environments focusing on viruses and microbial composition

Reference	Location	Equipment	Pathogen	Positivity
[207]	Hospital ward	Exposed plate; impinger and air sampler with a gelatin filter	Microbial composition	
[208]	Hospital ward	Solid impactor	Influenza virus	65%
[209]	Hospital; laboratory; student dorm; hotel room; dining hall	Cascade impactor with agar plates	Microbial composition	
[210]	Hospital ward and kitchen	Impactor with agar	Microbial composition	
[211]	Healthcare facility	Liquid impinger Cyclone	Norovirus	75%
[212]	Hospital operating theater; emergency service; surgical ward	Air sampler with agar plates	Aerobic mesophilic bacterial counts and fungal load	
[213]	Hospital ward	Cyclone and gelatin filter	MERS-CoV	57%
[214]	Hospital ward	Solid impactor and PTFE filter	Influenza A	80%
[215]	Hospital emergency room	Solid impactor and PTFE filter	Influenza A, influenza D, and adenovirus	27%
[216]	Emergency department	Air sampler with PTFE filter	Influenza	43%
[217]	acute care facility	Two-stage cyclone	Influenza virus	37%, 50%
[218]	Hospital ward	Air sampler with agar plates	Fungi	
[219]	Hospital ward	Petri plates with medium	Bacterial and fungal bioaerosols	
[220]	Hospital ward	Two-stage cyclone	Influenza A and Influenza B	79%, 20%
[221]	Healthcare facility	Air sampler with agar	Microbial composition	
[222]	Hospital outpatient hall, clinical laboratory, fever clinic, children’s ward	Impinger	Influenza (IFVA and IFVB)	
[223]	Hospital ward	Cyclone samplers	SARS-CoV-2	19%
[224]	Hospital wards and isolation rooms	Cyclone samplers	SARS-CoV-2	0.4% and 5%
[225]	Hospital ward and ICU	Cyclone samplers	SARS-CoV-2	7.7% and 82.6%
[226]	Hospital ward	Cyclone samplers	SARS-CoV-2	1.50%
[140]	Hospital ward	Cyclone samplers	SARS-CoV-2	67%
[227]	Hospital air outlets, ward and doctors’ room	Liquid impinger	SARS-CoV-2	35.7%, 44.4%, 12.5%
[228]	Hospital ICU	Liquid impinger	SARS-CoV-2	50%
[229]	Hospital ward	Liquid impinger	SARS-CoV-2	14%
[230]	Hotel toilet, corridor, room; Hospital ward ICU	Liquid impinger	SARS-CoV-2	10%, 0%
[231]	Hospital ward	Gelatin filter	SARS-CoV-2	100%
[205]	Hospital ward and personal items	Gelatin filter	SARS-CoV-2	58% and 100%
[232]	Hospital ward	Solid impactor; gelatin filter; liquid impactor	SARS-CoV-2	2%
[233]	Hospital ward and laboratory and rooms	Natural sedimentation	SARS-CoV-2	5%, 0%, 0%
[234]	Hospital ward	Water–vapor condensation	SARS-CoV-2	80%
[128]	Hospital ward; stuff area; public area	Solid impactor and deposition	SARS-CoV-2	64%, 77%, 44%
[235]	Hospital sites	Liquid cyclone	SARS-CoV-2	45%
[236]	Hospital waiting room	Cyclone sampler	Influenza A, B, parainfluenza 2, 3, human metapneumovirus, respiratory syncytial virus, human adenovirus, human bocavirus, and Bordetella pertussis	
[237]	Hospital ward	Liquid impinger Cyclone	Influenza virus and respiratory syncytial virus	51%
[238]	Hospital ICU	Cascade impactor	Airborne bacteria	
[239]	Hospital	Novel electrostatic collector	SARS-CoV-2	41% and 86%
[240]	Hospital	Cyclone and gelatin filter	SARS-CoV-2 and bacteria	0% and 7.3%
[241]	Hospital, isolation, and open-cohort wards		SARS-CoV-2	60–87%
[242]	Hospital ward	Cyclone bioaerosol sampler	SARS-CoV-2	3–7%
[243]	Hospital rooms and community isolation facilities	Water–vapor condensation method	SARS-CoV-2	50% and 11%
[244]	Hospital ICU and adjacent wards	PTFE membrane filters + Low volume sampler	SARS-CoV-2	12%, 50%
[245]	Hospital ward and ICU	Liquid impinger and cyclone with gelatin filter	SARS-CoV-2	0% and 83.3%
[246]	Hospital ward	Cellulose filter	SARS-CoV-2 and bacteria in parallel	30% and bacteria did not exceed the cleaning standards in the analyzed samples
[247]	Hospital various sites	Specialized gas wash bottle sampler	SARS-CoV-2, other respiratory viruses, and pathogenic bacteria parallel	
[248]	Geriatric ward	Glass fiber filter	SARS-CoV-2	100%
[249]	Nursing home	Dust filters	SARS-CoV-2	37–80%
[250]	Hospital ICU and ward; long-term care facility	Cyclone and gelatin filter	SARS-CoV-2	17%, 8%, 20%
[251]	Hospital ward, nurse station, toilet	Midget impinger	SARS-CoV-2	2.50%
[252]	Hospital ward and home	Dry samplers with PTFE filter parallel liquid impinger	SARS-CoV-2	0% and 50% (dry) / 0% (wet)
[201]	Hospital wards	Cascade impactor	Bacteria and fungi	
[253]	Hospital ward	Liquid impinger Cyclone with gelatin filter	SARS-CoV-2	50% and 11%
[254]	Hospital ward and nurse station	Liquid impinger	SARS-CoV-2	
[204]	Hospital and home care environments	Cascade impactor	SARS-CoV-2	45.2%
[88]	Hospital ward	Cascade impactor	SARS-CoV-2	59.2%

## Understanding the spread of the particles

Once we know the origin and the properties—e.g., the size distribution—of the potential pathogen carrier particles, the next step is to determine how they spread in and between rooms and closed or open compartments. Besides the simulations with CFD [144] and other methods [145], different groups also carried out measurements with tracer gas, samplers, or sensor networks and determined the hot spots, e.g., in theaters and auditorium halls [146], in an aeroplane cabin [147], or in bars and restaurants [148, 149].

In addition to the sedimentation and evaporation, the way (e.g., velocity, direction, environmental airflow field) the particles are emitted during coughing, sneezing, or other aerosol-generating actions is also quite important regarding the trajectory of the aerosol particles in the air. Thus, the fluid dynamics of the exhaled particle clouds are also widely investigated (e.g., [150, 151]). Many studies indicated that the jets with high velocity in case of sneezing or coughing are frequently capable of carrying the emitted aerosol particles quite far, so 1.5 m social distancing is frequently not enough to prevent infection.

The 1.5–2 m (or 6 foot) distance from the emitter is only effective against the large droplets that fall fast and are emitted with low velocities. During sneezing, relatively high air velocities can occur. For example, Han et al. [152], using high-frequency image particle velocimetry, measured the emitted particle’s velocity during coughing and sneezing. According to their results, the maximum instantaneous velocity was 15.6 m/s for females and 16.2 m/s for males. According to Netz [62], the time how long a particle with a diameter less than 50 µm remains in the air is quite long, more than 10 s, even if no evaporation is considered. With the initial velocity of 15–16 m/s, these particles may travel much more than 1.5 m. In addition, when sneezing and coughing, many small (with diameter less than 5 µm) particles are emitted, which can stay in the air for minutes or even hours. They can travel large distances with the local air flow. According to Gorbunov [153], particles may fly quite far (over 30 m) if there is airflow, such as wind. Based on the above considerations, no general rule for social distancing can be established. The situation must be assessed in each case, and the necessary protective measures must be taken accordingly.

In most of the models that aim to describe the behavior of the droplets in the air, it is not possible to appropriately simulate the effect of the complex airflow distribution in the environment. CFD methods offer a solution to this problem. By simulating the fluid dynamics of the particles on a mesh, the fine-scale pathway of the emitted droplets can be determined. Mohamadi and Fazeli presented a review of the applications of CFD modeling in the COVID-19 pandemic [154]. The conclusion of their work was that the current outdoor social distancing could not protect individuals from COVID-19 because of the impact of wind.

In tight spaces with complex airflow fields, CFD simulations are extremely useful in modeling the pathway of aerosol particles in the air. For example, [155] investigated the infection probability for passengers sharing the same cabin with an infected person for 30 min. Different scenarios were used to evaluate the effect of the infected subjects’ position within the car cabin and the usage of the heating, ventilation, and air conditioning (HVAC) system. One of their main findings was that the risk of infection in indoor environments could, in principle, be reduced by designing the airflows properly.

Similarly, using CFD simulations, [156] investigated the dispersion of cough-generated droplets in comparison with those emitted during talking and breathing during different activities in a cabin of an aeroplane. They found that the inhaled virus carried by droplet nuclei smaller than 10 μm accounted for more than 99% of the total amount of inhaled viruses.

The above review of the literature confirms that determining the pathway of the droplets emitted from the respiratory tract is a complex task influenced by both the environmental conditions, like airflow, humidity, and temperature, and the highly variable modes of emission (e.g., talking, coughing, sneezing).

Although different models give slightly different results, they all agree that large droplets fall down fast, but small particles may stay in the air for a considerable amount of time. If the ventilation rate is not sufficient, these particles can play an important role in pathogen transmission, even at large distances. This clearly indicates that besides social distancing, face masks have to be used to decrease the probability of infection.

Understanding the properties of respiratory particles and how they spread can also help us to remove them from the air. In the next section, we overview the air filtration techniques from this perspective.

## Air filtration and disinfection

In the context of spreading different contagious diseases, a healthy indoor environment based on air quality improvement and purification technologies has received increasing attention. Therefore, air quality control has become a critically important aspect of public health. To reduce the risk of infection, besides air filtration, many methodological approaches, like heat sterilization, chemical disinfectant, ventilation, and ultraviolet (UV) irradiation, can be used [157].

### Mechanical and electrostatic air filtration

Among the diverse methods of air purification, the most common technique is filtration, which involves the removal of airborne particles by filtering media. Several types of materials have been proposed for air filters, including membranes and fibrous materials [158]. These materials are assessed by various performance metrics, of which filtration efficiency is an essential factor that reflects the efficiency at which particles are removed. Additionally, the pressure drop is also important, indicating the amount of energy needed to purify polluted air. Based on these criteria, fibrous materials are the most widely used due to their highly porous structures that facilitate airflow while successfully removing particles [159, 160].

At the same time, using fibrous filters assumes the fulfilment of several controversial requirements. These filters typically have small pores and large thicknesses that provide high filtration efficiencies but substantial pressure drops, resulting in poor filtration performance. So, considerable efforts have been made to overcome this trade-off relationship in two ways: through structure- and interaction-based approaches. Basic filtration theory outlines several types of filtration mechanisms for particle capture: diffusion, interception, impaction, gravitational settling, and electrostatic attraction [160, 161]. All methods except for the electrostatic attraction are categorized as mechanical filtration mechanisms and largely depend on the structure of the air filter to physically remove particles by interfering with the laminar airflows that pass through the material. At the same time, electrostatic attraction predominantly exploits electrostatic interactions between particles and filtration media rather than altering the airstream. Thus, optimizing the filter structure and improving electrostatic interactions can balance filtration efficiency and pressure drop and further improve filtration performance. A comprehensive understanding of the structure and electrostatic interactions on filtration characteristics is expected to yield fundamental solutions to realize high-performance air filters.

Many existing personal filtering materials lack air permeability, even though numerous technological advances have focused on relieving pressure drops. This disadvantage is especially important for commercial face masks, which can be difficult for individuals with respiratory difficulties or in situations requiring intense physical movement. Therefore, innovative techniques should be further explored to enable adequate breathability of face masks. Second, managing large quantities of disposable filter media can become a significant societal burden. This issue arises because many air filter media irreversibly lose filtration capabilities due to their progressive ineffectiveness over time against humidity, chemicals, and mechanical stresses [162]. Thus, more effort should be devoted to developing reusable air filters, which would result in substantial cost savings and waste reduction that would help protect the planet. Third, the mechanical properties of filters still require improvement to achieve comfortable and effective personal protective equipment (PPE). In the case of the COVID-19 pandemic, PPE has become an essential part of daily life, and among a variety of PPE, face masks are considered the most effective means to protect people from getting or spreading the viral agent. However, their poor softness, flexibility, and stretchability impose several issues on users, such as skin irritation, bad wearing sensation, and loose fitting, which reduces their medical effectiveness because the gap between mouth and mask may cause leakage and virus spread to the ambient air. Though advanced filtration materials with fine flexibility and softness have been studied [163, 164], most of them are unsuitable for direct PPE application. Therefore, future studies should focus on the mechanical properties of filtration materials besides filtration performances, considering their applicability to other types of PPE. Finally, filtration materials with antipathogenic properties should be investigated to prevent the spread of viruses in filter media. Conventionally, metal-based materials have been incorporated into filtration materials for this purpose because even small amounts of metals, such as copper, gold, and silver, have biocidal activity, which is referred to as the oligodynamic effect [165, 166]. The ability to inactivate viral agents has yet to be completely verified, despite the fact that metals effectively eliminate other pathogens, such as bacteria, fungi, and so on. Thus, exploring advanced capturing and inactivating mechanisms of nanosized pathogens, such as viruses, is urgently needed to address the current pandemic and prepare for the next one [167].

### Disinfection by UV-C radiation

UV-based disinfection has become a common chemical-free technology in the last decades [168]. The UV-C light with wavelength 200–280 nm can be effectively absorbed by the biomolecules, i.e., nucleic acid or proteins, leading to the generation of photoproducts that inactivate the pathogens. Thanks to this, it is very effective in disinfecting surfaces, air, and liquids [169].

During the COVID-19 pandemic, the survival of SARS-COV-2 after UV irradiation was investigated in many studies. For example, Heilingloh et al. irradiated SARS-CoV-2 culture in a liquid medium and found that after 9 min of exposure, a significant amount of the virus was destroyed [170]. In another study, far UV-C light was used to reduce surface contamination of SARS-COV-2. They found that 222 nm UV-C light reduced the number of viable viruses by 99.7% in 30 s [171]. Gidari et al. showed that UV-C irradiation of surfaces efficiently reduced virus titer (99.99%), with doses ranging from 10.25 to 23.71 mJ/cm^2^ [86]. Beggs and Avital reported that UV-C light at wavelengths of about 254 nm exerts bactericidal and virucidal effects, and it is widely used in the environmental disinfection of enclosed spaces [172].

UV-C irradiation can be used to deactivate coronaviruses in the aerosol particles floating in the air, too. Walker and Ko suggested 0.7 mJ cm^−2^ for 90% inactivation of coronavirus-laden aerosols [173]. This result agrees with the outcome of earlier studies by Wiess and Horzinek [174] and Hirano et al. [175], as well as those reported by Saknimit et al. [176]. Different pathogens and even different strains of the same virus can require different exposure to UV light.

For air disinfection, in-duct UV systems are commonly adopted. They consist of arrays of UV bulbs installed in air ventilation ducts to inactivate pathogens in moving air streams [177]. In addition, more and more autonomous disinfection systems like UV robots are on the market [178]. These are capable of inactivating the pathogens without the risk of unwanted UV exposure.

For disinfection purposes, a variety of UV-C sources can be used, including low and medium-pressure mercury UV lamps [179], UV light-emitting diodes (UV-LEDs) [180], and far-UV-C (200–240 nm) radiating excimer and microplasma lamps [181].

UV-C radiation can damage skin and eyes, so only properly designed systems should be used where direct contact with the workers with the radiation is minimized. In addition, ozone can be generated, which can irritate the airways. Wavelengths below 240 nm more readily generate ozone [182]. Reducing the ozone generated by these UV lamps can be achieved by enveloping the lamp with material (for example, specific soft glass) that is only transparent for wavelengths longer than 240 nm).

## Risk assessment and maintaining safe environments

### Risk assessment

It is already well known that human infections are caused by air transmission of aerosolized pathogens; thus to make a valuable risk assessment, it is crucial to have an adequate amount and quality of data about infectious airborne particles on site and information about emitters [183–185] as well as numerical models that are well-suited to simulate the near-field evolution of respiratory particles. Accordingly, much research has been done to determine the adequate sampling and analytical methods (see Table 4) to define the most critical events and factors which may have potential hazards or risks in hospitals [87, 186, 187] and to develop an appropriate infection risk model [188–202] for various pathogens. However, especially in the case of Coronavirus, sampling methods seem to be still inadequate mainly due to the lack of effective control and mitigation measurements and the fact that the commonly used RT-PCR has limitations as it may quantify non-infectious viruses [143, 203]. Additionally, the results of [202] suggest that the transmission dynamics vary for different viruses in healthcare settings, which suggests a prominent role in case studies.

### Maintaining a safe environment in healthcare and nursing-care settings

Given the considerations above, when designing a healthcare or nursing-care environment, knowing the size-distribution and quantity of infectious particles and assessing their dynamics and the characteristics of pathogens is vital. Besides the pathogenicity, the most important characteristics of the infectious agents are their biological lifetime and the time particles spend in the air.

As seen in the section describing sampling methods for indoor aerosol and pathogens and Table 4, many measurement results are available on air pollution with pathogens in healthcare facilities. While most of the samples were limited to pathogen detection, in some cases, their distribution was also determined in different size ranges [88, 128, 204]. In a recent study [66], optical particle counters were utilized to perform real-life measurements of size-fractionated aerosol concentration in a plethysmography box, and the associated viral load was estimated using mathematical methods. The authors found that patients emit the same amount of particles during an examination as they do during normal speech, so the risk of hospital staff performing active breathing maneuvers with the patients becoming infected during the test is not higher than when talking to the same patient.

Optical particle counters were also used in a study to examine the particles generated by various activities performed by healthcare professionals caring for patients in a real-world hospital setting [87]. The authors found that in the nursing home bedroom, staff and patient activities significantly increased the concentration of submicron particles in the air. In contrast, the concentration of supermicron particles increased mainly during nursing activities. While submicron particles mainly deposit in the acinar regions, the supermicron particles deposit in the larger airways. This information became crucial when it was shown that, in addition to particles 1–5 microns in size, viral RNA was detected on particles in the submicron range and even below 300 nm [88, 128].

There are different ways to remove particles containing pathogens from the air. In addition to various filtration techniques, airflow design or dilution of the particle concentration (ventilation) can remove particles from the local environment, but the neutralization of pathogens is also a viable option.

High-efficiency particulate air (HEPA) filters remove larger particles from the air by sieving and inertial impaction and smaller particles by interception and diffusion. While the filtration efficiency of HEPA filters is close to 100% for larger particles (above 0.5–1 µm) and smaller particles (below 100–50 nm), it is slightly lower in between, with a minimum in the range of 100–500 nm, which should be taken into account in light of the fact that pathogens can be detected on particles in this range.

In some cases, a negative pressure environment was developed to protect the health care professionals from infection [205], while in other cases, a positive pressure environment was created to protect severely immunocompromised patients [206].

### Time periods of increased precaution

The question of how long to wait after a medical procedure or other activity resulting in increased aerosol formation before it is safe to enter the room became particularly relevant during the COVID pandemic (especially when PPE was in reduced supply). Infection control at multiple institutions mandating time periods of increased precaution after medical procedures that might represent increased production of droplets (bronchoscopy and intubation).

A solid particle’s settling time is inversely proportional to the square of its radius (the bigger the particle, the less time it spends in the air). In contrast to solid particles, pure water particles do evaporate. If a particle is very small, it may evaporate before settling down. On the other hand, very big particles can fall down before they evaporate. Water particles large enough not to evaporate rapidly but small enough not to fall quickly spend the most time in the air. The initial size of the particles with the longest floating time depends very much on the relative humidity but falls in the range of a few tens of micrometers, and their floating time is a few seconds.

However, exhaled particles are neither solid nor water particles. The particles emitted from the respiratory tract usually contain non-volatile content, too. For instance, these can be organic macromolecules, pathogens, and even hydration water, which, mostly due to electrostatic interactions, does not evaporate but forms the so-called hydration shell around the charged molecules. Moreover, salts (e.g., sodium chloride and calcium) may also be present in the exhaled aerosol particles, attracting more water. Therefore, the evaporation time can be higher for these particles than for pure water droplets. At a given time after their exhalation, exhaled particles tend to have bigger sizes than their pure water counterparts due to their non-volatile fraction, slower evaporation, or even hygroscopic behavior. As a bigger size is associated with a shorter settling time, exhaled particles float for a shorter time. Actually, if we compare the values in Table 1 (water particles) with the values in Table 3 (particles with 1% non-volatile fraction), we observe that assuming saturated air (RH = 1), a water particle of 1 µm settles in 16.6 h, while a particle with the same initial size but with 1% non-volatile fraction settles in 5 h.

Now, in terms of risk, it is hard to assess whether the modified behavior of particles due to their non-volatile content will increase or decrease the risk. The particle size of the most lingering particles with non-volatile content is under 1 µm, so much smaller than it was in the case of water particles. Assuming that pathogen content is proportional to the volume leads to the conclusion that fewer floating pathogens are in the air. However, the floating time of these particles is much higher than that of the most lingering water particles, suggesting an accumulation of pathogens in the air. This also suggests a longer waiting time between two aerosol-generating medical interventions (e.g., bronchoscopy).

As discussed before, due to the complicated air flow patterns and heterogeneous conditions in a room, no general rule can be set for the waiting time between treatments in medical diagnostic rooms. In each case, it depends on the room and its ventilation system. If the air exchange rate for a particular diagnostic room is known, the time for the particulate concentration to fall below a certain level can be determined.

### Research-informed practical suggestions

We can summerize the following guidelines and practical steps for individual patients and clinicians to emphasize the effectiveness of certain interventions and mitigation strategies:

- Regular monitoring of air quality is crucial in healthcare and nursing facilities, especially in measuring the levels of particulate matter and pathogen-laden aerosols, as there is a correlation between particulate matter (especially PM_2.5_) concentration and respiratory tract infections. This helps clinicians promptly identify potential health risks and adjust infection control protocols accordingly.
- Aerosol generation activities such as normal speaking can generate approximately 50 aerosol particles per second, while louder activities such as singing or shouting can increase this number by a factor of 10. During a sneeze, as many as 20,000 droplets can be expelled, travelling at speeds exceeding 20 m/s. Conversely, coughing produces 10 to 100 times fewer particles, with a velocity of approximately 10 m/s. In confined healthcare or geriatric environments, these activities can significantly raise the concentration of airborne particles. Properly worn masks, particularly those designed with multi-layered materials, can reduce aerosol transmission by over 90% under controlled conditions. Encouraging mask usage in high-risk environments can significantly decrease the probability of pathogen spread.
- Specific medical interventions, like bronchoscopy or spirometry, can produce many aerosol particles, posing risks of airborne transmission. Introducing of more strict safety protocols for aerosol-generating procedures, especially during peak infection periods, can mitigate infection risks.
- Enclosed, poorly ventilated spaces exacerbate the risk of aerosol spread. For instance, aerosolized particles smaller than 5 microns can remain airborne in poorly ventilated rooms for up to several hours. This increases the risk of inhaling pathogen-laden aerosols in environments like geriatric care units, where vulnerable individuals are present. More emphasis on disinfection measures should be made in facilities with high aerosol-generating procedures, including bronchoscopy, lung function, or rehabilitation rooms.
- High-efficiency particulate air (HEPA) filters can capture up to 99.97% of airborne particles that are 0.3 microns in diameter or larger. This range is significant for pathogen-carrying particles, as many respiratory aerosols fall within this size. Regular maintenance of these filters is necessary to ensure that they continue to operate at optimal efficiency, especially in enclosed healthcare spaces where PM_2.5_ particles, capable of carrying viruses, can linger. Studies show that even after being filtered, particles can be reintroduced into the environment, necessitating a layered approach to filtration, ventilation, and personal protection.
- Localized air purification systems, such as portable HEPA filters, can be used in areas where it is difficult to implement full-room ventilation. These units can be placed strategically to maximize the removal of pathogens near the source. Studies have shown that placing such units near aerosol-generating activities can reduce airborne particle concentrations by up to 90% within a short radius.
- Since submicron particles can float in the air for days, only ventilation, air filtration, and disinfection can reduce the waiting time between medical procedures in medical diagnostic rooms. Proper ventilation can reduce airborne pathogen concentrations by diluting contaminated air. Studies show that increasing the air exchange rate in enclosed spaces can significantly lower aerosol concentration. For example, increasing air changes per hour (ACH) from 3 to 12 in a healthcare setting can decrease the concentration of aerosols by more than 80%. This underlines the importance of implementing high ACH rates, particularly in critical care areas like geriatric wards.
- While HEPA filters are highly effective at removing particles, regular air movements within rooms, such as patient transfers, can resuspend particles into the air. This emphasizes the importance of minimizing unnecessary movement in enclosed spaces and implementing airflow controls to limit particle resuspension during high-risk periods. Research indicates that reducing high-aerosol activities in enclosed spaces can lower the airborne particle count by up to 80%, significantly lowering the risk of airborne transmission.
- Effective airflow management ensures that clean air is directed towards patient areas and that contaminated air is exhausted efficiently. In some studies, improper ventilation flow patterns in confined spaces, such as hospital rooms, have been shown to increase the risk of aerosol transmission. Optimizing airflow direction—ensuring that contaminated air is directed away from patients and staff—can reduce cross-contamination risks.
- Negative pressure rooms are essential in isolating airborne infections by ensuring that contaminated air does not escape into other areas. These rooms can maintain a constant airflow inward, preventing pathogens from spreading to adjoining spaces. Conversely, positive pressure rooms can protect immunocompromised patients by ensuring that only filtered air enters the room, keeping pathogens out.

## Conclusions

This comprehensive review has explored the recent literature on the measurement and simulation of processes related to exhalation, transport, filtration, inhalation, and airway deposition of pathogen-carrying aerosol particles. The COVID-19 pandemic has sparked unprecedented research in this area, resulting in several key insights with particular relevance to geriatric populations.

We found that all enclosed or specially ventilated rooms, including healthcare facilities and nursing homes, contain significant amounts of PM_2.5_ particles. Normal respiratory activities such as breathing, coughing, sneezing, and talking generate aerosols capable of carrying pathogens. Although novel methods for measuring the RNA or DNA of pathogens have enhanced diagnostic capabilities, preserving the viability of small aerosol samples remains a challenge. Mechanical filtration systems, such as HEPA filters, effectively reduce PM_2.5_ particles; however, normal movements within a room can stir these particles, potentially resuspending them into the air. Submicron particles remain airborne for extended periods and can penetrate deeply into the human respiratory system, reaching the alveolar regions and potentially entering the bloodstream, thus posing significant health risks.

The elderly, particularly those in geriatric care settings, are at a heightened risk from these airborne pathogens. The vulnerability of older adults is compounded by factors such as weakened immune systems, the presence of chronic conditions, and the communal nature of long-term care facilities. This makes it imperative to understand and control aerosol transmission to protect this demographic.

The deposition of inhaled particles can be modeled for individuals with specific breathing patterns, aiding in understanding individual susceptibility and exposure risks. Environmental factors significantly influence air quality in healthcare settings, necessitating regular air quality measurements. For older adults, who often have pre-existing respiratory conditions, the impact of air quality is even more pronounced, making these measurements crucial.

Despite these advancements, several areas require further investigation to enhance our understanding and control of airborne pathogen transmission in geriatric settings. Improved methods for sampling small aerosol particles that preserve pathogen viability need to be developed. Detailed studies on the resuspension of particles in indoor environments and the effectiveness of different mitigation strategies are essential. Enhanced computational models that integrate a wider range of variables, including individual health status, activity levels, and environmental conditions, are necessary. Longitudinal studies on the impact of chronic exposure to PM_2.5_ and other aerosols on older adults should be conducted. Additionally, exploring air quality control and infection prevention measures in extreme environments, such as low-gravity or zero-G spaces, could provide insights applicable to terrestrial settings.

The findings from this review offer several practical recommendations for improving air quality and infection control in healthcare and geriatric settings. Implementing advanced mechanical filtration systems, such as HEPA filters, in all enclosed spaces within healthcare and nursing facilities is crucial. Ensuring regular maintenance and checks will maximize their efficiency. Routine air quality assessments in healthcare environments are necessary to identify and mitigate potential risks promptly. Encouraging the use of personal protective equipment (PPE), especially masks, among healthcare workers and patients can significantly reduce the transmission of airborne pathogens. Managing and possibly limiting activities that generate high levels of aerosols in enclosed spaces is particularly important during periods of high infection risk. Designing and implementing tailored airflow systems in critical areas such as isolation wards and geriatric care units will help minimize the spread of infectious aerosols. Providing ongoing education and training for healthcare professionals on the latest findings in aerosol transmission and effective mitigation strategies is essential. Adopting a holistic approach to infection control that considers individual patient factors, environmental conditions, and the dynamic nature of aerosol behavior will further enhance safety.

In conclusion, while significant strides have been made in understanding and mitigating airborne pathogen transmission, ongoing research and the implementation of practical measures are essential to protect vulnerable populations, particularly older adults in healthcare and geriatric settings. The continuous improvement of air quality and infection control practices will be crucial in reducing morbidity and mortality associated with respiratory pathogens, thus ensuring better health outcomes for the elderly.

## References

[1] Centers for Disease Control and Prevention: Provisional Death Counts for Coronavirus Disease (COVID-19) [Internet]. [cited 2024 Jun 23]. Available from: https://www.cdc.gov/nchs/nvss/vsrr/covid19/index.htm

[2] Peterfi A, Meszaros A, Szarvas Z, Penzes M, Fekete M, Feher A, et al. Comorbidities and increased mortality of COVID-19 among the elderly: a systematic review. Physiol Int. 2022. 10.1556/2060.2022.00206 35575986

[3] Fekete M, Szarvas Z, Fazekas-Pongor V, Feher A, Dosa N, Lehoczki A, et al. COVID-19 infection in patients with chronic obstructive pulmonary disease: from pathophysiology to therapy. Physiol Int: Mini-review; 2022. 10.1556/2060.2022.00172 35230261

[4] Feher A, Szarvas Z, Lehoczki A, Fekete M, Fazekas-Pongor V. Co-infections in COVID-19 patients and correlation with mortality rate. Physiol Int: Mini-review; 2022. 10.1556/2060.2022.00015 35218335

[5] Ferioli M, Cisternino C, Leo V, Pisani L, Palange P, Nava S. Protecting healthcare workers from sars-cov-2 infection: practical indications. Eur Respir Rev. 2020;29(155):1–10. 10.1183/16000617.0068-2020 PMC7134482 32248146

[6] Meyerowitz EA, Richterman A, Gandhi RT, Sax PE. Transmission of SARS-CoV-2: a review of viral, host, and environmental factors. Ann Intern Med. 2021;174:69–79. 32941052 10.7326/M20-5008 PMC7505025

[7] Edwards DA, Ausiello D, Salzman J, Devlin T, Langer R, Beddingfield BJ, et al. Exhaled aerosol increases with COVID-19 infection, age, and obesity. Proc Natl Acad Sci. 2021;118(8):e2021830118. 33563754 10.1073/pnas.2021830118 PMC7923364

[8] Szulc J, Okrasa M, Ryngajllo M, Pielech-Przybylska K, Gutarowska B. Markers of chemical and microbiological contamination of the air in the sport centers. Molecules. 2023;28(8):3560. 37110794 10.3390/molecules28083560 PMC10144153

[9] Sayahi T, Workman AD, Kelly KE, Ardon-Dryer K, Presto AA, Bleier BS. Aerosol generation during nasal airway instrumentation. Otolaryngol Head Neck Surg. 2023;168:506–13. 35503253 10.1177/01945998221099028

[10] Russo F, Valentini M, Sabatino D, Cerati M, Facco C, Battaglia P, et al. Aerosolization risk during endoscopic transnasal surgery: a prospective qualitative and quantitative microscopic analysis of particles spreading in the operating room. J Neurosurg. 2022;136:822–30. 34534965 10.3171/2021.3.JNS204415

[11] Hamilton FW, Gregson FKA, Arnold DT, Sheikh S, Ward K, Brown J, et al. Aerosol emission from the respiratory tract: an analysis of aerosol generation from oxygen delivery systems. Thorax. 2022;77:276–82. 34737195 10.1136/thoraxjnl-2021-217577 PMC8867281

[12] Nikitin N, Petrova E, Trifonova E, Karpova O. Influenza virus aerosols in the air and their infectiousness. Adv Virol. 2014;859090. 10.1155/2014/859090 PMC4147198 25197278

[13] Le Sage V, Lowen AC, Lakdawala SS. Block the spread: barriers to transmission of influenza viruses. Annu Rev Virol. 2023;10:347–70. 37308086 10.1146/annurev-virology-111821-115447

[14] Killingley B, Nguyen-Van-Tam J. Routes of influenza transmission. Influenza Respir Viruses. 2013;7(Suppl 2):42–51. 10.1111/irv.12080 PMC5909391 24034483

[15] Hall CB. Nosocomial respiratory syncytial virus infections: the ‘Cold War’ has not ended. Clin Infect Dis. 2000;31:590–6. 10987726 10.1086/313960

[16] Coleman M, Martinez L, Theron G, Wood R, Marais B. Mycobacterium tuberculosis transmission in high-incidence settings-new paradigms and insights. Pathogens. 2022;11(11):1228. 36364978 10.3390/pathogens11111228 PMC9695830

[17] Herfst S, Bohringer M, Karo B, Lawrence P, Lewis NS, Mina MJ, et al. Drivers of airborne human-to-human pathogen transmission. Curr Opin Virol. 2017;22:22–9. 27918958 10.1016/j.coviro.2016.11.006 PMC7102691

[18] Nel A. Air pollution-related illness: effects of particles. Science. 2005;308(5723):804–6. 15879201 10.1126/science.1108752

[19] Timonen KL, Vanninen E, de Hartog J, Ibald-Mulli A, Brunekreef B, Gold DR, et al. Effects of ultrafine and fine particulate and gaseous air pollution on cardiac autonomic control in subjects with coronary artery disease: the ULTRA study. J Expo Sci Environ Epidemiol. 2006;16(4):332–41. 16205787 10.1038/sj.jea.7500460

[20] Brook RD, Rajagopalan S, Pope CA, Brook JR, Bhatnagar A, Diez-Roux AV, et al. Particulate matter air pollution and cardiovascular disease. Circulation. 2010;121(21):2331–78. 20458016 10.1161/CIR.0b013e3181dbece1

[21] Wu X, Nethery RC, Sabath MB, Braun D, Dominici F. Air pollution and COVID-19 mortality in the United States: strengths and limitations of an ecological regression analysis. Sci Adv. 2020;6(45):ead4049. 10.1126/sciadv.abd4049 PMC7673673 33148655

[22] Cui Y, Zhang ZF, Froines J, Zhao J, Wang H, Yu SZ, et al. Air pollution and case fatality of SARS in the People’s Republic of China: an ecologic study. Environ Health. 2003;2(1):15. 14629774 10.1186/1476-069X-2-15 PMC293432

[23] Ciencewicki J, Jaspers I. Air pollution and respiratory viral infection. Inhal Toxicol. 2007;19(14):1135–46. 17987465 10.1080/08958370701665434

[24] Chen PS, Tsai FT, Lin CK, Yang CY, Chan CC, Young CY, et al. Ambient influenza and avian influenza virus during dust storm days and background days. Environ Health Perspect. 2010;118(9):1211–6. 20435545 10.1289/ehp.0901782 PMC2944079

[25] Després VivianeR, Huffman JA, Burrows SM, Hoose C, Safatov AleksandrS, Buryak G, et al. Primary biological aerosol particles in the atmosphere: a review. Tellus B Chem Phys Meteorol. 2012 Jan 1;64(1):15598.

[26] Vandini S, Corvaglia L, Alessandroni R, Aquilano G, Marsico C, Spinelli M, et al. Respiratory syncytial virus infection in infants and correlation with meteorological factors and air pollutants. Ital J Pediatr. 2013;39(1):1. 23311474 10.1186/1824-7288-39-1 PMC3553040

[27] Ye Q, Fu J fen, Mao J hua, Shang S qiang. Haze is a risk factor contributing to the rapid spread of respiratory syncytial virus in children. Environ Sci Pollut Res. 2016 Oct 1;23(20):20178–85. 10.1007/s11356-016-7228-6 27439752

[28] Nenna R, Evangelisti M, Frassanito A, Scagnolari C, Pierangeli A, Antonelli G, et al. Respiratory syncytial virus bronchiolitis, weather conditions and air pollution in an Italian urban area: an observational study. Environ Res. 2017;1(158):188–93. 10.1016/j.envres.2017.06.014 PMC7125886 28647513

[29] Peng L, Zhao X, Tao Y, Mi S, Huang J, Zhang Q. The effects of air pollution and meteorological factors on measles cases in Lanzhou. China Environ Sci Pollut Res. 2020;27(12):13524–33. 10.1007/s11356-020-07903-4 32030582

[30] Setti L, Passarini F, De Gennaro G, Barbieri P, Perrone MG, Borelli M, et al. SARS-Cov-2RNA found on particulate matter of Bergamo in Northern Italy: first evidence. Environ Res. 2020;188:109754. 32526492 10.1016/j.envres.2020.109754 PMC7260575

[31] Nikolich-Zugich J, Knox KS, Rios CT, Natt B, Bhattacharya D, Fain MJ. SARS-CoV-2 and COVID-19 in older adults: what we may expect regarding pathogenesis, immune responses, and outcomes. GeroScience. 2020;42(2):505–14. 32274617 10.1007/s11357-020-00186-0 PMC7145538

[32] Lee WJ, Chen LK, Tang GJ, Lan TY. The impact of influenza vaccination on hospitalizations and mortality among frail older people. J Am Med Dir Assoc. 2014;15:256–60. 24559640 10.1016/j.jamda.2013.12.003

[33] Hartman L, Zhu Y, Edwards KM, Griffin MR, Talbot HK. Underdiagnosis of influenza virus infection in hospitalized older adults. J Am Geriatr Soc. 2018;66:467–72. 29341100 10.1111/jgs.15298 PMC5863754

[34] Chan TC, Hung IFN, Luk JKH, Chu LW, Chan FHW. Effectiveness of influenza vaccination in institutionalized older adults: a systematic review. J Am Med Dir Assoc. 2014;15(226):1–266. 24321878 10.1016/j.jamda.2013.10.008

[35] Metcalf CJE, Paireau J, O’Driscoll M, Pivette M, Hubert B, Pontais I, et al. Comparing the age and sex trajectories of SARS-CoV-2 morbidity and mortality with other respiratory pathogens. R Soc Open Sci. 2022;9:211498. 35719888 10.1098/rsos.211498 PMC9198511

[36] Palacios-Pedrero MA, Jansen JM, Blume C, Stanislawski N, Jonczyk R, Molle A, et al. Signs of immunosenescence correlate with poor outcome of mRNA COVID-19 vaccination in older adults. Nat Aging. 2022;2:896–905. 37118289 10.1038/s43587-022-00292-y PMC10154205

[37] Reber AJ, Chirkova T, Kim JH, Cao W, Biber R, Shay DK, et al. Immunosenescence and challenges of vaccination against influenza in the aging population. Aging Dis. 2012;3:68–90. 22500272 PMC3320806

[38] Carrazana E, Ruiz-Gil T, Fujiyoshi S, Tanaka D, Noda J, Maruyama F, et al. Potential airborne human pathogens: a relevant inhabitant in built environments but not considered in indoor air quality standards. Sci Total Environ. 2023;25(901):165879. 10.1016/j.scitotenv.2023.165879 37517716

[39] Wells WF. On air-borne infection: Study II. Droplets and droplet nuclei. Am J Epidemiol. 1934;20(3):611–8.

[40] Jennison MW. Atomizing of mouth and nose secretions into the air as revealed by high speed photography. Aerobiology. 1942;17:106–28.

[41] Duguid JP. The numbers and the sites of origin of the droplets expelled during expiratory activities. Edinb Med J. 1945;LII(II):385–401. PMC5286249 21009905

[42] Duguid JP. The size and the duration of air-carriage of respiratory droplets and droplet-nuclei. J Hyg (Lond). 1946;44(6):471–9. 20475760 10.1017/s0022172400019288 PMC2234804

[43] Loudon RG, Roberts RM. Singing and the dissemination of tuberculosis. Am Rev Respir Dis. 1968;98(2):297–300. 5667756 10.1164/arrd.1968.98.2.297

[44] Loudon RG, Roberts RM. Droplet expulsion from the respiratory tract. Am Rev Respir Dis. 1967;95(3):435–42. 6018703 10.1164/arrd.1967.95.3.435

[45] Fairchild CI, Stampfer JF. Particle concentration in exhaled breath. Am Ind Hyg Assoc J. 1987;48(11):948–9. 3425555 10.1080/15298668791385868

[46] Papineni RS, Rosenthal FS. The size distribution of droplets in the exhaled breath of healthy human subjects. 1997;10(2):105–16. 10.1089/jam.1997.10.105 10168531

[47] Yang S, Lee GWM, Chen CM, Wu CC, Yu KP. The size and concentration of droplets generated by coughing in human subjects. J Aerosol Med-Depos Clear Eff Lung. 2007;20(4):484–94. 10.1089/jam.2007.0610 18158720

[48] Lindsley WG, Noti JD, Blachere FM, Thewlis RE, Martin SB, Othumpangat S, et al. Viable influenza A virus in airborne particles from human coughs. J Occup Environ Hyg. 2015;12(2):107–13. 25523206 10.1080/15459624.2014.973113 PMC4734406

[49] Dbouk T, Drikakis D. On respiratory droplets and face masks. Phys Fluids. 2020;32(6):063303. 10.1063/5.0015044 PMC7301882 32574231

[50] Feng Y, Marchal T, Sperry T, Yi H. Influence of wind and relative humidity on the social distancing effectiveness to prevent COVID-19 airborne transmission: a numerical study. J Aerosol Sci. 2020;1(147):105585. 10.1016/j.jaerosci.2020.105585 PMC7233256 32427227

[51] Wang H, Li Z, Zhang X, Zhu L, Liu Y, Wang S. The motion of respiratory droplets produced by coughing. Phys Fluids. 2020;32(12):125102. 10.1063/5.0033849 PMC7757605 33362402

[52] Mittal R, Ni R, Seo JH. The flow physics of COVID-19. J Fluid Mech. 2020;894:F2.

[53] Johnson GR, Morawska L, Ristovski ZD, Hargreaves M, Mengersen K, Chao CYH, et al. Modality of human expired aerosol size distributions. J Aerosol Sci. 2011;42(12):839–51. 10.1016/j.jaerosci.2008.10.003 PMC7126899 32287373

[54] Lindsley WG, Pearce TA, Hudnall JB, Davis KA, Davis SM, Fisher MA, et al. Quantity and size distribution of cough-generated aerosol particles produced by influenza patients during and after illness. J Occup Environ Hyg. 2012;9(7):443–9. 22651099 10.1080/15459624.2012.684582 PMC4676262

[55] Coleman KK, Tay DJW, Tan KS, Ong SWX, Than TS, Koh MH, et al. Viral load of severe acute respiratory syndrome coronavirus 2 (SARS-CoV-2) in respiratory aerosols emitted by patients with coronavirus disease 2019 (COVID-19) while breathing, talking, and singing. Clin Infect Dis Off Publ Infect Dis Soc Am. 2022;74(10):1722–8. 10.1093/cid/ciab691 PMC8436389 34358292

[56] Stockman T, Zhu S, Kumar A, Wang L, Patel S, Weaver J, et al. Measurements and simulations of aerosol released while singing and playing wind instruments. ACS Environ Au. 2021;1(1):71–84. 37155479 10.1021/acsenvironau.1c00007 PMC8525345

[57] Sheikh S, Hamilton FW, Nava GW, Gregson FKA, Arnold DT, Riley C, et al. Are aerosols generated during lung function testing in patients and healthy volunteers? Results from the AERATOR study Thorax. 2022;77(3):292–4. 34728573 10.1136/thoraxjnl-2021-217671

[58] Morawska L, Johnson GR, Ristovski ZD, Hargreaves M, Mengersen K, Corbett S, et al. Size distribution and sites of origin of droplets expelled from the human respiratory tract during expiratory activities. J Aerosol Sci. 2009;40(3):256–69. 10.1016/j.jaerosci.2008.10.003 PMC7126899 32287373

[59] Holmgren H, Ljungström E, Almstrand AC, Bake B, Olin AC. Size distribution of exhaled particles in the range from 0.01 to 2.0 μm. J Aerosol Sci. 2010 May 1;41(5):439–46.

[60] Gregson FKA, Watson NA, Orton CM, Haddrell AE, McCarthy LP, Finnie TJR, et al. Comparing aerosol concentrations and particle size distributions generated by singing, speaking and breathing. Aerosol Sci Technol. 2021;55(6):681–91.

[61] Wang CC, Prather KA, Sznitman J, Jimenez JL, Lakdawala SS, Tufekci Z, et al. Airborne transmission of respiratory viruses. Science. 2021;373(6558):eabd9149. 34446582 10.1126/science.abd9149 PMC8721651

[62] Netz RR. Mechanisms of airborne infection via evaporating and sedimenting droplets produced by speaking. J Phys Chem B. 2020;124(33):7093–101. 32668904 10.1021/acs.jpcb.0c05229 PMC7409921

[63] WHO. Transmission of SARS-CoV-2: implications for infection prevention precautions [Internet]. 2020 [cited 2023 Feb 2]. Available from: https://www.who.int/news-room/commentaries/detail/transmission-of-sars-cov-2-implications-for-infection-prevention-precautions

[64] Helgeson SA, Lim KG, Lee AS, Niven AS, Patel NM. Aerosol generation during spirometry. Ann Am Thorac Soc. 2020;17(12):1637–9. 32870021 10.1513/AnnalsATS.202005-569RL PMC7706605

[65] Milanese M, Corsico AG, Bellofiore S, Carrozzi L, Di Marco F, Iovene B, et al. Suggestions for lung function testing in the context of COVID-19. Respir Med. 2021;1(177):106292. 10.1016/j.rmed.2020.106292 PMC7773526 33440299

[66] Tomisa G, Horváth A, Farkas Á, Nagy A, Kis E, Tamási L. Real-life measurement of size-fractionated aerosol concentration in a plethysmography box during the COVID-19 pandemic and estimation of the associated viral load. J Hosp Infect. 2021;1(118):7–14. 10.1016/j.jhin.2021.08.025 PMC8414843 34487775

[67] Greening NJ, Larsson P, Ljungström E, Siddiqui S, Olin AC. Small droplet emission in exhaled breath during different breathing manoeuvres: implications for clinical lung function testing during COVID-19. Allergy. 2021;76(3):915–7. 32966612 10.1111/all.14596 PMC7537081

[68] Li J, Jing G, Fink JB, Porszasz J, Moran EM, Kiourkas RD, et al. Airborne particulate concentrations during and after pulmonary function testing. Chest. 2021;159(4):1570–4. 33144080 10.1016/j.chest.2020.10.064 PMC8807333

[69] WHO. Coronavirus disease (COVID-19): How is it transmitted? [Internet]. 2021 [cited 2023 Jun 8]. Available from: https://www.who.int/news-room/questions-and-answers/item/coronavirus-disease-covid-19-how-is-it-transmitted

[70] WHO. Modes of transmission of virus causing COVID-19: implications for IPC precaution recommendations [Internet]. 2020 [cited 2023 Jun 8]. Available from: https://www.who.int/news-room/commentaries/detail/modes-of-transmission-of-virus-causing-covid-19-implications-for-ipc-precaution-recommendations

[71] Lewis D. Mounting evidence suggests coronavirus is airborne — but health advice has not caught up. Nature. 2020;583(7817):510–3. 32647382 10.1038/d41586-020-02058-1

[72] Greenhalgh T, Jimenez JL, Prather KA, Tufekci Z, Fisman D, Schooley R. Ten scientific reasons in support of airborne transmission of SARS-CoV-2. The Lancet. 2021;397(10285):1603–5. 10.1016/S0140-6736(21)00869-2 PMC8049599 33865497

[73] Asadi S, Wexler AS, Cappa CD, Barreda S, Bouvier NM, Ristenpart WD. Aerosol emission and superemission during human speech increase with voice loudness. Sci Rep. 2019;9(1):2348. 30787335 10.1038/s41598-019-38808-z PMC6382806

[74] Alsved M, Matamis A, Bohlin R, Richter M, Bengtsson PE, Fraenkel CJ, et al. Exhaled respiratory particles during singing and talking. Aerosol Sci Technol. 2020;54(11):1245–8.

[75] Fabian P, Brain J, Houseman EA, Gern J, Milton DK. Origin of exhaled breath particles from healthy and human rhinovirus-infected subjects. J Aerosol Med Pulm Drug Deliv. 2011;24(3):137–47. 21361786 10.1089/jamp.2010.0815 PMC3123971

[76] Johnson GR, Morawska L. The mechanism of breath aerosol formation. J Aerosol Med Pulm Drug Deliv. 2009;22(3):229–37. 19415984 10.1089/jamp.2008.0720

[77] Abkarian M, Stone HA. Stretching and break-up of saliva filaments during speech: a route for pathogen aerosolization and its potential mitigation. Phys Rev Fluids. 2020;5(10): 102301.

[78] Rodríguez-Hakim M, Räz L, Vermant J. Variations in human saliva viscoelasticity affect aerosolization propensity. Soft Matter. 2022;18(13):2528–40. 35113119 10.1039/d1sm01581h

[79] Bake B, Larsson P, Ljungkvist G, Ljungström E, Olin AC. Exhaled particles and small airways. Respir Res. 2019;20(1):1–14. 30634967 10.1186/s12931-019-0970-9 PMC6330423

[80] Rezaei M, Netz RR. Airborne virus transmission via respiratory droplets: effects of droplet evaporation and sedimentation. Curr Opin Colloid Interface Sci. 2021;1(55): 101471. 10.1016/j.cocis.2021.101471 PMC8164513 34093064

[81] de Oliveira PM, Mesquita LCC, Gkantonas S, Giusti A, Mastorakos E. Evolution of spray and aerosol from respiratory releases: theoretical estimates for insight on viral transmission. Proc R Soc Math Phys Eng Sci. 2021;477(2245):20200584. 10.1098/rspa.2020.0584 PMC7897643 33633490

[82] Nicas M, Nazaroff WW, Hubbard A. Toward understanding the risk of secondary airborne infection: emission of respirable pathogens. J Occup Environ Hyg. 2005;2(3):143. 15764538 10.1080/15459620590918466 PMC7196697

[83] Chen W, Zhang N, Wei J, Yen HL, Li Y. Short-range airborne route dominates exposure of respiratory infection during close contact. Build Environ. 2020;1(176):106859.

[84] DasSarma P, DasSarma S. Survival of microbes in Earth’s stratosphere. Curr Opin Microbiol. 2018;1(43):24–30. 10.1016/j.mib.2017.11.002 29156444

[85] van Doremalen N, Bushmaker T, Morris DH, Holbrook MG, Gamble A, Williamson BN, et al. Aerosol and surface stability of SARS-CoV-2 as compared with SARS-CoV-1. N Engl J Med. 2020;382(16):1564–7. 32182409 10.1056/NEJMc2004973 PMC7121658

[86] Gidari A, Sabbatini S, Bastianelli S, Pierucci S, Busti C, Bartolini D, et al. SARS-CoV-2 Survival on surfaces and the effect of UV-C light. Viruses. 2021;13(3):408. 33807521 10.3390/v13030408 PMC7998261

[87] Nagy A, Horváth A, Farkas Á, Füri P, Erdélyi T, Madas BG, et al. Modeling of nursing care-associated airborne transmission of SARS-CoV-2 in a real-world hospital setting. GeroScience. 2022;44(2):585–95. 34985588 10.1007/s11357-021-00512-0 PMC8729098

[88] Groma V, Kugler S, Farkas Á, Füri P, Madas B, Nagy A, et al. Size distribution and relationship of airborne SARS-CoV-2 RNA to indoor aerosol in hospital ward environments. Sci Rep. 2023;13(1):3566. 36864124 10.1038/s41598-023-30702-z PMC9980870

[89] Gehr P, Heyder J, editors. Particle-lung interactions. New York: Marcel Dekker Inc; 2000. 823 p. (Lung biology in health and disease (exec. ed. Lenfant C); vol. 143).

[90] Ruzer LS, Harley NH, editors. Aerosols handbook: measurement, dosimetry, and health effects, Second Edition. 2nd ed. Boca Raton: CRC Press; 2012. 666 p.

[91] GINA. Global Initiative for Asthma - GINA. 2021 [cited 2023 Feb 2]. GINA, 2021. (Global Initiative for Asthma) Global strategy for asthma management and prevention. Available from: https://ginasthma.org/gina-reports/

[92] GOLD. Global Initiative for Chronic Obstructive Lung Disease - GOLD. 2021 [cited 2023 Feb 2]. GOLD, 2021. (Global Initiative for Chronic Obstructive Lung Disease) Global strategy for prevention, diagnosis and management of COPD. Available from: https://goldcopd.org/archived-reports/

[93] Ye Y, Ma Y, Zhu J. The future of dry powder inhaled therapy: promising or discouraging for systemic disorders? Int J Pharm. 2022;25(614):121457. 10.1016/j.ijpharm.2022.121457 PMC8744475 35026316

[94] ICRP. Human Respiratory Tract Model for Radiological Protection. ICRP Publication 66. Ann. ICRP 24 (1–3). Pergamon Press, Oxford, UK. 1994. 7726471

[95] Findeisen W. Über das Absetzen kleiner, in der Luft suspendierter Teilchen in der menschlichen Lunge bei der Atmung. Pflüg Arch Für Gesamte Physiol Menschen Tiere. 1935;236(1):367–79.

[96] Landahl HD. On the removal of air-borne droplets by the human respiratory tract: I. The lung Bull Math Biophys. 1950;12(1):43–56.

[97] Beeckmans JM. Correction factor for size-selective sampling results, based on a new computed alveolar deposition curve. Ann Occup Hyg. 1965;8(3):221–31. 14328235 10.1093/annhyg/8.3.221

[98] Bolch WE, Farfán EB, Huh C, Huston TE, Bolch WE. Influences of parameter uncertainties within the ICRP 66 respiratory tract model: particle deposition. Health Phys. 2001;81(4):378. 11569633 10.1097/00004032-200110000-00003

[99] Cassee FR, Freijer JI, Subramaniam R, Asgharian B, Miller FJ, van Bree L, et al. Development of a model for human and rat airway particle deposition: implications for risk assessment. RIVM report 650010 018. [Internet]. RIJKSINSTITUUT VOOR VOLKSGEZONDHEID EN MILIEU NATIONAL INSTITUTE OF PUBLIC HEALTH AND THE ENVIRONMENT; 1999. Available from: https://rivm.openrepository.com/bitstream/handle/10029/10229/650010018.pdf?sequence=1&isAllowed=y

[100] Hofmann W, Koblinger L. Monte Carlo modeling of aerosol deposition in human lungs. Part III: comparison with experimental data. J Aerosol Sci. 1992 Jan 1;23(1):51–63.

[101] Hofmann W, Koblinger L. Monte Carlo modeling of aerosol deposition in human lungs. Part II: deposition fractions and their sensitivity to parameter variations. J Aerosol Sci. 1990;21(5):675–88.

[102] Koblinger L, Hofmann W. Monte Carlo modeling of aerosol deposition in human lungs. Part I: simulation of particle transport in a stochastic lung structure. J Aerosol Sci. 1990;21(5):661–74.

[103] Koblinger L, Hofmann W. Analysis of human lung morphometric data for stochastic aerosol deposition calculations. Phys Med Biol. 1985;30(6):541–56. 4011676 10.1088/0031-9155/30/6/004

[104] Raabe OG, Yeah HC, Schum MG, Phalen RF. UNT Digital Library. United States. Department of Commerce.; 1976 [cited 2023 Feb 3]. Tracheobronchial geometry: human, dog, rat, hamster - a compilation of selected data from the project respiratory tract deposition models. Available from: https://digital.library.unt.edu/ark:/67531/metadc100754/

[105] Farkas A, Jo´kay A, Fu¨ri P, Bala´sha´zy I, Mu¨ller V, Odler B, et al. Computer modelling as a tool in characterization and optimization of aerosol drug delivery. Aerosol Air Qual Res. 2015;15(6):2466–74.

[106] Füri P, Groma V, Török S, Farkas Á, Dian C. Ultrafine urban particle measurements in Budapest and their airway deposition distribution calculation. Inhal Toxicol. 2020;32(13–14):494–502. 33283557 10.1080/08958378.2020.1850937

[107] Madas BG, Füri P, Farkas Á, Nagy A, Czitrovszky A, Balásházy I, et al. Deposition distribution of the new coronavirus (SARS-CoV-2) in the human airways upon exposure to cough-generated droplets and aerosol particles. Sci Rep. 2020 Dec 1;10(1). 10.1038/s41598-020-79985-6 PMC7775446 33384436

[108] Füri P. Köhögéskor kibocsátott, kórokozó tartalmú cseppek légzõrendszeri kiülepedéseloszlása – az új típusú koronavírus (SARS-CoV-2) aeroszol formában történõ terjedése. Fiz Szle. 2021;71(4):117–21.

[109] Radhakrishnan H, Kassinos S. CFD modeling of turbulent flow and particle deposition in human lungs. Annu Int Conf IEEE Eng Med Biol Soc IEEE Eng Med Biol Soc Annu Int Conf. 2009;2009:2867–70. 10.1109/IEMBS.2009.5333102 19963784

[110] Johnson TJ, Irwin M, Symonds JPR, Olfert JS, Boies AM. Measuring aerosol size distributions with the aerodynamic aerosol classifier. Aerosol Sci Technol. 2018;52(6):655–65.

[111] Xie X, Li Y, Sun H, Liu L. Exhaled droplets due to talking and coughing. J R Soc Interface. 2009;6(suppl_6):S703-14. 19812073 10.1098/rsif.2009.0388.focus PMC2843952

[112] Chao CYH, Wan MP, Morawska L, Johnson GR, Ristovski ZD, Hargreaves M, et al. Characterization of expiration air jets and droplet size distributions immediately at the mouth opening. J Aerosol Sci. 2009;40(2):122–33. 32287373 10.1016/j.jaerosci.2008.10.003 PMC7126899

[113] Zhu S, Kato S, Yang JH. Study on transport characteristics of saliva droplets produced by coughing in a calm indoor environment. Build Environ. 2006;41(12):1691–702.

[114] Tang JW, Liebner TJ, Craven BA, Settles GS. A schlieren optical study of the human cough with and without wearing masks for aerosol infection control. J R Soc Interface. 2009;6(Suppl 6):S727-736. 19815575 10.1098/rsif.2009.0295.focus PMC2843945

[115] Kwon SB, Park J, Jang J, Cho Y, Park DS, Kim C, et al. Study on the initial velocity distribution of exhaled air from coughing and speaking. Chemosphere. 2012;87(11):1260–4. 22342283 10.1016/j.chemosphere.2012.01.032 PMC7112028

[116] Han ZY, Weng WG, Huang QY. Characterizations of particle size distribution of the droplets exhaled by sneeze. J R Soc Interface. 2013;10(88):20130560. 24026469 10.1098/rsif.2013.0560 PMC3785820

[117] Pan M, Lednicky J a, Wu CY. Collection, particle sizing and detection of airborne viruses. J Appl Microbiol. 2019;127(6):1596–611. 30974505 10.1111/jam.14278 PMC7167052

[118] Hogan C j, Kettleson E m, Lee MH, Ramaswami B, Angenent L t, Biswas P. Sampling methodologies and dosage assessment techniques for submicrometre and ultrafine virus aerosol particles. J Appl Microbiol. 2005;99(6):1422–34. 16313415 10.1111/j.1365-2672.2005.02720.x

[119] Cao G, Noti JD, Blachere FM, Lindsley WG, Beezhold DH. Development of an improved methodology to detect infectious airborne influenza virus using the NIOSH bioaerosol sampler. J Environ Monit. 2011;13(12):3321–8. 21975583 10.1039/c1em10607d PMC4820822

[120] Lednicky J, Pan M, Loeb J, Hsieh H, Eiguren-Fernandez A, Hering S, et al. Highly efficient collection of infectious pandemic influenza H1N1 virus (2009) through laminar-flow water based condensation. Aerosol Sci Technol. 2016;50(7):i–iv.

[121] Lindsley WG, Green BJ, Blachere FM, Martin SB, Jensen PA, Schafer MP. Sampling and characterization of bioaerosols. NIOSH Man Anal Methods NMAM 5th Ed [Internet] [cited 2023 Feb 2]. 2017;BA. Available from: https://www.cdc.gov/niosh/nmam/pdf/chapter-ba.pdf

[122] Verreault D, Moineau S, Duchaine C. Methods for sampling of airborne viruses. Microbiol Mol Biol Rev MMBR. 2008;72(3):413–44. 18772283 10.1128/MMBR.00002-08 PMC2546863

[123] Borges JT, Nakada LYK, Maniero MG, Guimarães JR. SARS-CoV-2: a systematic review of indoor air sampling for virus detection. Environ Sci Pollut Res. 2021;28(30):40460–73. 10.1007/s11356-021-13001-w PMC7905194 33630259

[124] Dinoi A, Feltracco M, Chirizzi D, Trabucco S, Conte M, Gregoris E, et al. A review on measurements of SARS-CoV-2 genetic material in air in outdoor and indoor environments: Implication for airborne transmission. Sci Total Environ. 2022;25(809):151137. 10.1016/j.scitotenv.2021.151137 PMC8539199 34699823

[125] Santarpia JL, Klug E, Ravnholdt A, Kinahan SM. Environmental sampling for disease surveillance: recent advances and recommendations for best practice. J Air Waste Manag Assoc. 2023;73(6):434–61. 37224401 10.1080/10962247.2023.2197825

[126] Bourgueil E, Hutet E, Cariolet R, Vannier P. Air sampling procedure for evaluation of viral excretion level by vaccinated pigs infected with Aujeszky’s disease (pseudorabies) virus. Res Vet Sci. 1992;52(2):182–6. 1316629 10.1016/0034-5288(92)90008-p

[127] Kenny LC, Thorpe A, Stacey P. A collection of experimental data for aerosol monitoring cyclones. Aerosol Sci Technol. 2017;51(10):1190–200.

[128] Liu Y, Ning Z, Chen Y, Guo M, Liu Y, Gali NK, et al. Aerodynamic analysis of SARS-CoV-2 in two Wuhan hospitals. Nature. 2020;582(7813):557–60. 32340022 10.1038/s41586-020-2271-3

[129] Lindsley WG, Blachere FM, Beezhold DH, Thewlis RE, Noorbakhsh B, Othumpangat S, et al. Viable influenza A virus in airborne particles expelled during coughs versus exhalations. Influenza Other Respir Viruses. 2016;10(5):404–13. 26991074 10.1111/irv.12390 PMC4947941

[130] Springorum AC, Clauß M, Hartung J. A temperature-controlled AGI-30 impinger for sampling of bioaerosols. Aerosol Sci Technol. 2011;45(10):1231–9.

[131] Fabian P, McDevitt JJ, Houseman EA, Milton DK. Airborne influenza virus detection with four aerosol samplers using molecular and infectivity assays: considerations for a new infectious virus aerosol sampler. Indoor Air. 2009;19(5):433–41. 19689447 10.1111/j.1600-0668.2009.00609.x PMC3684270

[132] Jang J, Akin D, Lim KS, Broyles S, Ladisch MR, Bashir R. Capture of airborne nanoparticles in swirling flows using non-uniform electrostatic fields for bio-sensor applications. Sens Actuators B Chem. 2007;121(2):560–6.

[133] Cheng L, Lan L, Ramalingam M, He J, Yang Y, Gao M, et al. A review of current effective COVID-19 testing methods and quality control. Arch Microbiol. 2023;205(6):239. 37195393 10.1007/s00203-023-03579-9 PMC10189236

[134] Corman VM, Landt O, Kaiser M, Molenkamp R, Meijer A, Chu DK, et al. Detection of 2019 novel coronavirus (2019-nCoV) by real-time RT-PCR. Eurosurveillance. 2020;25(3):2000045. 31992387 10.2807/1560-7917.ES.2020.25.3.2000045 PMC6988269

[135] Lu X, Wang L, Sakthivel SK, Whitaker B, Murray J, Kamili S, et al. US CDC real-time reverse transcription PCR panel for detection of severe acute respiratory syndrome coronavirus 2. Emerg Infect Dis [Internet]. 2020 [cited 2023 Sep 15];26(8). Available from: https://wwwnc.cdc.gov/eid/article/26/8/20-1246_article 10.3201/eid2608.201246 PMC7392423 32396505

[136] Sciuto EL, Leonardi AA, Calabrese G, Luca GD, Coniglio MA, Irrera A, et al. Nucleic acids analytical methods for viral infection diagnosis: state-of-the-art and future perspectives. Biomolecules. 2021;11(11):1585. 34827583 10.3390/biom11111585 PMC8615992

[137] Javalkote VS, Kancharla N, Bhadra B, Shukla M, Soni B, Sapre A, et al. CRISPR-based assays for rapid detection of SARS-CoV-2. Methods. 2022;1(203):594–603. 10.1016/j.ymeth.2020.10.003 PMC7546951 33045362

[138] Zhou Y, Zhang L, Xie YH, Wu J. Advancements in detection of SARS-CoV-2 infection for confronting COVID-19 pandemics. Lab Invest. 2022;102(1):4–13. 34497366 10.1038/s41374-021-00663-w PMC8424153

[139] Atoui A, Cordevant C, Chesnot T, Gassilloud B. SARS-CoV-2 in the environment: contamination routes, detection methods, persistence and removal in wastewater treatment plants. Sci Total Environ. 2023;10(881):163453. 10.1016/j.scitotenv.2023.163453 PMC10091716 37059142

[140] Chia PY, Coleman KK, Tan YK, Ong SWX, Gum M, Lau SK, et al. Detection of air and surface contamination by SARS-CoV-2 in hospital rooms of infected patients. Nat Commun. 2020;11(1):2800. 32472043 10.1038/s41467-020-16670-2 PMC7260225

[141] Mihajlovski K, Buttner MP, Cruz P, Labus B, Schneider BSP, Detrick E. SARS-CoV-2 surveillance with environmental surface sampling in public areas. PLoS ONE. 2022;17(11):e0278061. 36417446 10.1371/journal.pone.0278061 PMC9683569

[142] Rozman U, Knez L, Novak G, Golob J, Pulko A, Cimerman M, et al. Environmental contamination with SARS-CoV-2 in hospital COVID department: antigen test, real-time RT-PCR and virus isolation. COVID. 2022;2(8):1050–6.

[143] Yun H, Yang J, Seo JH, Sohn JR. Methodology for sampling and detection of airborne coronavirus including SARS-CoV-2. Indoor Built Environ. 2022;31(5):1234–41.

[144] van Beest MRRS, Arpino F, Hlinka O, Sauret E, van Beest NRTP, Humphries RS, et al. Influence of indoor airflow on particle spread of a single breath and cough in enclosures: does opening a window really ‘help’? Atmospheric Pollut Res. 2022;13(7):101473. 10.1016/j.apr.2022.101473 PMC9167821 35692900

[145] Endo A, CMMID COVID-19 Working Group, Uchida (内田満夫) M, Liu (刘扬) Y, Atkins KE, Kucharski AJ, et al. Simulating respiratory disease transmission within and between classrooms to assess pandemic management strategies at schools. Proc Natl Acad Sci. 2022 Sep 13;119(37):e2203019119. 10.1073/pnas.2203019119 PMC9478679 36074818

[146] Adzic F, Roberts BM, Hathway EA, Kaur Matharu R, Ciric L, Wild O, et al. A post-occupancy study of ventilation effectiveness from high-resolution CO2 monitoring at live theatre events to mitigate airborne transmission of SARS-CoV-2. Build Environ. 2022;1(223):109392. 10.1016/j.buildenv.2022.109392 PMC9339161 35937085

[147] Hertzberg VS, Weiss H, Elon L, Si W, Norris SL, The FlyHealthy Research Team. Behaviors, movements, and transmission of droplet-mediated respiratory diseases during transcontinental airline flights. Proc Natl Acad Sci. 2018 Apr 3;115(14):3623–7. 10.1073/pnas.1711611115 PMC5889623 29555754

[148] Li Y, Qian H, Hang J, Chen X, Cheng P, Ling H, et al. Probable airborne transmission of SARS-CoV-2 in a poorly ventilated restaurant. Build Environ. 2021;1(196):107788. 10.1016/j.buildenv.2021.107788 PMC7954773 33746341

[149] Van der Veer BMJW, Gorgels KMF, den Heijer CDJ, Hackert V, van Alphen LB, Savelkoul PHM, Hoebe CJPA and Dingemans J. SARS-CoV-2 transmission dynamics in bars, restaurants, and nightclubs. Front Microbiol. 2023;14:1183877. 10.3389/fmicb.2023.1183877 PMC10232797 37275153

[150] Bourouiba L, Dehandschoewercker E, Bush JWM. Violent expiratory events: on coughing and sneezing. J Fluid Mech. 2014;745:537–63.

[151] Xie X, Li Y, Chwang ATY, Ho PL, Seto WH. How far droplets can move in indoor environments – revisiting the Wells evaporation–falling curve. Indoor Air. 2007;17(3):211–25. 17542834 10.1111/j.1600-0668.2007.00469.x

[152] Han M, Ooka R, Kikumoto H, Oh W, Bu Y, Hu S. Experimental measurements of airflow features and velocity distribution exhaled from sneeze and speech using particle image velocimetry. Build Environ. 2021;1(205):108293. 10.1016/j.buildenv.2021.108293 PMC8663001 34908645

[153] Gorbunov B. Aerosol particles generated by coughing and sneezing of a SARS-CoV-2 (COVID-19) host travel over 30 m distance. Aerosol Air Qual Res. 2021;21(3):200468.

[154] Mohamadi F, Fazeli A. A review on applications of CFD modeling in COVID-19 pandemic. Arch Comput Methods Eng. 2022;29(6):3567–86. 35079217 10.1007/s11831-021-09706-3 PMC8773396

[155] Arpino F, Grossi G, Cortellessa G, Mikszewski A, Morawska L, Buonanno G, et al. Risk of SARS-CoV-2 in a car cabin assessed through 3D CFD simulations. Indoor Air. 2022;32(3):e13012. 35347787 10.1111/ina.13012 PMC9111293

[156] Wang W, Wang F, Lai D, Chen Q. Evaluation of SARS-COV-2 transmission and infection in airliner cabins. Indoor Air. 2022;32(1):e12979. 35048429 10.1111/ina.12979

[157] Raeiszadeh M, Adeli B. A critical review on ultraviolet disinfection systems against COVID-19 outbreak: applicability, validation, and safety considerations. ACS Photonics. 2020;7(11):2941–51. 37556269 10.1021/acsphotonics.0c01245

[158] Hinds WC. Aerosol technology: properties, behavior, and measurement of airborne particles. New York: John Wiley & Sons; 1999. p. 150–4.

[159] Zhu M, Han J, Wang F, Shao W, Xiong R, Zhang Q, et al. Electrospun nanofibers membranes for effective air filtration. Macromol Mater Eng. 2017;302(1):1600353.

[160] Kadam VV, Wang L, Padhye R. Electrospun nanofibre materials to filter air pollutants – a review. J Ind Text. 2018;47(8):2253–80.

[161] Davies CN. Deposition of aerosols from turbulent flow through pipes. Proc R Soc Lond Ser Math Phys Sci. 1966;289(1417):235–46.

[162] Tabti B, Mekideche MR, Plopeanu MC, Dumitran LM, Antoniu A, Dascalescu L. Factors that influence the decay rate of the potential at the surface of nonwoven fabrics after negative corona discharge deposition. IEEE Trans Ind Appl. 2010;46(4):1586–92.

[163] Liu H, Zhang S, Liu L, Yu J, Ding B. A fluffy dual-network structured nanofiber/net filter enables high-efficiency air filtration. Adv Funct Mater. 2019;29(39):1904108.

[164] Zhang S, Liu H, Tang N, Zhou S, Yu J, Ding B. Spider-web-inspired PM0.3 filters based on self-sustained electrostatic nanostructured networks. Adv Mater. 2020;32(29):2002361. 32510646 10.1002/adma.202002361 PMC7300536

[165] Mazia D, Mullins LJ. Radioactive copper and the mechanism of oligodynamic action. Nature. 1941;147(3734):642–642.

[166] Prasher P, Singh M, Mudila H. Oligodynamic effect of silver nanoparticles: a review. BioNanoScience. 2018;8(4):951–62.

[167] Han S, Kim J, Ko SH. Advances in air filtration technologies: structure-based and interaction-based approaches. Mater Today Adv. 2021;1(9):100134.

[168] Kowalski W. Ultraviolet germicidal irradiation handbook: UVGI for air and surface disinfection [Internet]. Berlin, Heidelberg: Springer; 2009 [cited 2024 Sep 17]. Available from: 10.1007/978-3-642-01999-9

[169] Dai T, Vrahas MS, Murray CK, Hamblin MR. Ultraviolet C irradiation: an alternative antimicrobial approach to localized infections? Expert Rev Anti Infect Ther. 2012;10(2):185–95. 22339192 10.1586/eri.11.166 PMC3292282

[170] Heilingloh CS, Aufderhorst UW, Schipper L, Dittmer U, Witzke O, Yang D, et al. Susceptibility of SARS-CoV-2 to UV irradiation. Am J Infect Control. 2020;48(10):1273–5. 32763344 10.1016/j.ajic.2020.07.031 PMC7402275

[171] Kitagawa H, Nomura T, Nazmul T, Omori K, Shigemoto N, Sakaguchi T, et al. Effectiveness of 222-nm ultraviolet light on disinfecting SARS-CoV-2 surface contamination. Am J Infect Control. 2021;49(3):299–301. 32896604 10.1016/j.ajic.2020.08.022 PMC7473342

[172] Beggs CB, Avital EJ. Upper-room ultraviolet air disinfection might help to reduce COVID-19 transmission in buildings: a feasibility study. PeerJ. 2020;13(8): e10196. 10.7717/peerj.10196 PMC7566754 33083158

[173] Walker CM, Ko G. Effect of ultraviolet germicidal irradiation on viral aerosols. Environ Sci Technol. 2007;41(15):5460–5. 17822117 10.1021/es070056u

[174] Weiss M, Horzinek MC. Resistance of Berne virus to physical and chemical treatment. Vet Microbiol. 1986;11(1):41–9. 3518225 10.1016/0378-1135(86)90005-2 PMC7117442

[175] Hirano N, Hino S, Fujiwara K. Physico-chemical properties of mouse hepatitis virus (MHV-2) grown on DBT cell culture. Microbiol Immunol. 1978;22(7):377–90. 30881 10.1111/j.1348-0421.1978.tb00384.x PMC7168437

[176] Saknimit M, Inatsuki I, Sugiyama Y, Yagami K ichi. Virucidal efficacy of physico-chemical treatments against coronaviruses and parvoviruses of laboratory animals. Exp Anim. 1988;37(3):341–5. 10.1538/expanim1978.37.3_341 3416941

[177] Nunayon SS, Zhang H, Lai ACK. Comparison of disinfection performance of UVC-LED and conventional upper-room UVGI systems. Indoor Air. 2020;30(1):180–91. 31688980 10.1111/ina.12619

[178] Murphy RR, Gandudi VBM, Amin T, Clendenin A, Moats J. An analysis of international use of robots for COVID-19. Robot Auton Syst. 2022;1(148):103922. 10.1016/j.robot.2021.103922 PMC8591979 34803220

[179] Craik SA, Weldon D, Finch GR, Bolton JR, Belosevic M. Inactivation of *cryptosporidium parvum* oocysts using medium- and low-pressure ultraviolet radiation. Water Res. 2001;35(6):1387–98. 11317885 10.1016/s0043-1354(00)00399-7

[180] Song K, Taghipour F, Mohseni M. Microorganisms inactivation by wavelength combinations of ultraviolet light-emitting diodes (UV-LEDs). Sci Total Environ. 2019;15(665):1103–10. 10.1016/j.scitotenv.2019.02.041 30893742

[181] Raeiszadeh M, Taghipour F. Microplasma UV lamp as a new source for UV-induced water treatment: protocols for characterization and kinetic study. Water Res. 2019;1(164):114959. 10.1016/j.watres.2019.114959 31415967

[182] Claus H. Ozone generation by ultraviolet lamps. Photochem Photobiol. 2021;97(3):471–6. 33534912 10.1111/php.13391

[183] Hewlett AL, Whitney SE, Gibbs SG, Smith PW, Viljoen HJ. Mathematical modeling of pathogen trajectory in a patient care environment. Infect Control Hosp Epidemiol. 2013;34(11):1181–8. 24113602 10.1086/673451

[184] Jones RM, Brosseau LM. Aerosol transmission of infectious disease. J Occup Environ Med. 2015;57(5):501. 25816216 10.1097/JOM.0000000000000448

[185] Duchaine C, Roy CJ. Bioaerosols and airborne transmission: integrating biological complexity into our perspective. Sci Total Environ. 2022;15(825):154117. 10.1016/j.scitotenv.2022.154117 PMC8865953 35218821

[186] Almeida SM, Sousa J. Modelling the contribution of factors influencing the risk of SARS-CoV-2 infection in indoor environments. Acta Médica Port. 2021;34(12):815–25. 10.20344/amp.15982 34748475

[187] Kiersnowska ZM, Lemiech-Mirowska E, Sierocka A, Zawadzki M, Michałkiewicz M, Marczak M. Application of a novel PM model to assess the risk of Clostridioides difficile infections in medical facilities as a tool for improving the quality of health services and the safety of patients. Int J Environ Res Public Health. 2022;19(1):441. 10.3390/ijerph19010441 PMC8744772 35010698

[188] Han. IAENG Conference Publications: proceedings book of the world congress on engineering 2014 Vol. II. In 2014 [cited 2023 Feb 2]. Available from: https://www.iaeng.org/publication/details/WCE2014_Proc_II.html

[189] You S, Wan MP. A risk assessment scheme of infection transmission indoors incorporating the impact of resuspension. Risk Anal. 2015;35(8):1488–502. 25808677 10.1111/risa.12350

[190] Carducci A, Donzelli G, Cioni L, Verani M. Quantitative microbial risk assessment in occupational settings applied to the airborne human adenovirus infection. Int J Environ Res Public Health. 2016;13(7):733. 27447658 10.3390/ijerph13070733 PMC4962274

[191] Adhikari U, Chabrelie A, Weir M, Boehnke K, McKenzie E, Ikner L, et al. A case study evaluating the risk of infection from middle eastern respiratory syndrome coronavirus (MERS-CoV) in a hospital setting through bioaerosols. Risk Anal. 2019;39(12):2608–24. 31524301 10.1111/risa.13389 PMC7169172

[192] Fierce L, Robey AJ, Hamilton C. Simulating near-field enhancement in transmission of airborne viruses with a quadrature-based model. Indoor Air. 2021;31(6):1843–59. 34297863 10.1111/ina.12900 PMC8447483

[193] Hussein T, Löndahl J, Thuresson S, Alsved M, Al-Hunaiti A, Saksela K, et al. Indoor model simulation for COVID-19 transport and exposure. Int J Environ Res Public Health. 2021;18(6):2927. 33809366 10.3390/ijerph18062927 PMC7999367

[194] Nordsiek F, Bodenschatz E, Bagheri G. Risk assessment for airborne disease transmission by poly-pathogen aerosols. PLoS ONE. 2021;16(4):e0248004. 33831003 10.1371/journal.pone.0248004 PMC8031403

[195] Jones B, Sharpe P, Iddon C, Hathway EA, Noakes CJ, Fitzgerald S. Modelling uncertainty in the relative risk of exposure to the SARS-CoV-2 virus by airborne aerosol transmission in well mixed indoor air. Build Environ. 2021;15(191):107617. 10.1016/j.buildenv.2021.107617 PMC7816614 33495667

[196] Li X, Lester D, Rosengarten G, Aboltins C, Patel M, Cole I. A spatiotemporally resolved infection risk model for airborne transmission of COVID-19 variants in indoor spaces. Sci Total Environ. 2022;15(812):152592. 10.1016/j.scitotenv.2021.152592 PMC8695516 34954184

[197] Auvinen M, Kuula J, Grönholm T, Sühring M, Hellsten A. High-resolution large-eddy simulation of indoor turbulence and its effect on airborne transmission of respiratory pathogens—model validation and infection probability analysis. Phys Fluids. 2022;34(1):015124. 10.1063/5.0076495 PMC8939551 35340682

[198] Riediker M, Briceno-Ayala L, Ichihara G, Albani D, Poffet D, Tsai DH, et al. Higher viral load and infectivity increase risk of aerosol transmission for Delta and Omicron variants of SARS-CoV-2. Swiss Med Wkly. 2022;152(0102):w30133–w30133. 35019196 10.4414/smw.2022.w30133

[199] WU Jialin WW. Effect of human movement on a patient’s exhaled viral particle transmission: a numerical study. J Tsinghua Univ Technol. 2022 May 6;62(6):1044–51.

[200] Xu C, Liu W, Luo X, Huang X, Nielsen PV. Prediction and control of aerosol transmission of SARS-CoV-2 in ventilated context: from source to receptor. Sustain Cities Soc. 2022;1(76):103416. 10.1016/j.scs.2021.103416 PMC8484231 34611508

[201] Yousefzadeh A, Maleki A, Athar SD, Darvishi E, Ahmadi M, Mohammadi E, et al. Evaluation of bio-aerosols type, density, and modeling of dispersion in inside and outside of different wards of educational hospital. Environ Sci Pollut Res. 2022;29(10):14143–57. 10.1007/s11356-021-16733-x PMC8487404 34601681

[202] Zhang X, Wu J, Smith LM, Li X, Yancey O, Franzblau A, et al. Monitoring SARS-CoV-2 in air and on surfaces and estimating infection risk in buildings and buses on a university campus. J Expo Sci Environ Epidemiol. 2022;32(5):751–8. 35477766 10.1038/s41370-022-00442-9 PMC9045468

[203] Duval D, Palmer JC, Tudge I, Pearce-Smith N, O’Connell E, Bennett A, et al. Long distance airborne transmission of SARS-CoV-2: rapid systematic review. BMJ. 2022;29(377):e068743. 10.1136/bmj-2021-068743 PMC9240778 35768139

[204] Cvitešić Kušan A, Baranašić J, Frka S, Lucijanić T, Šribar A, Knežević J, et al. The size distribution of SARS-CoV-2 genetic material in airborne particles sampled in hospital and home care environments occupied by COVID-19 positive subjects. Sci Total Environ. 2023;20(892):164642. 10.1016/j.scitotenv.2023.164642 PMC10238117 37271394

[205] Santarpia JL, Rivera DN, Herrera VL, Morwitzer MJ, Creager HM, Santarpia GW, et al. Aerosol and surface contamination of SARS-CoV-2 observed in quarantine and isolation care. Sci Rep. 2020;10(1):12732. 32728118 10.1038/s41598-020-69286-3 PMC7391640

[206] Qian H, Zheng X. Ventilation control for airborne transmission of human exhaled bio-aerosols in buildings. J Thorac Dis. 2018;10(Suppl 19):S2295–304. 30116608 10.21037/jtd.2018.01.24 PMC6072925

[207] Sudharsanam S, Swaminathan S, Ramalingam A, Thangavel G, Annamalai R, Steinberg R, et al. Characterization of indoor bioaerosols from a hospital ward in a tropical setting. Afr Health Sci. 2012;12(2):217–25. 23056031 10.4314/ahs.v12i2.22 PMC3462539

[208] Bischoff WE, Swett K, Leng I, Peters TR. Exposure to influenza virus aerosols during routine patient care. J Infect Dis. 2013;207(7):1037–46. 23372182 10.1093/infdis/jis773

[209] Xu Z, Yao M. Monitoring of bioaerosol inhalation risks in different environments using a six-stage Andersen sampler and the PCR-DGGE method. Environ Monit Assess. 2013;185(5):3993–4003. 22955887 10.1007/s10661-012-2844-1

[210] Setlhare G, Malebo N, Shale K, Lues R. Identification of airborne microbiota in selected areas in a health-care setting in South Africa. BMC Microbiol. 2014;14(1):100. 24750818 10.1186/1471-2180-14-100 PMC4016773

[211] Bonifait L, Charlebois R, Vimont A, Turgeon N, Veillette M, Longtin Y, et al. Detection and quantification of airborne norovirus during outbreaks in healthcare facilities. Clin Infect Dis Off Publ Infect Dis Soc Am. 2015;61(3):299–304. 10.1093/cid/civ321 25900175

[212] Verde S, Almeida SM, Matos J, Guerreiro D, Meneses M, Faria T, et al. Microbiological assessment of indoor air quality at different hospital sites. Res Microbiol. 2015;166(7):557–63. 25869221 10.1016/j.resmic.2015.03.004

[213] Kim SH, Chang SY, Sung M, Park JH, Bin Kim H, Lee H, et al. Extensive viable middle east respiratory syndrome (MERS) coronavirus contamination in air and surrounding environment in MERS isolation wards. Clin Infect Dis. 2016;63(3):363–9. 27090992 10.1093/cid/ciw239 PMC7108054

[214] Leung NHL, Zhou J, Chu DKW, Yu H, Lindsley WG, Beezhold DH, et al. Quantification of influenza virus RNA in aerosols in patient rooms. PLoS ONE. 2016;11(2):e0148669. 26849130 10.1371/journal.pone.0148669 PMC4743992

[215] Choi JY, Zemke J, Philo SE, Bailey ES, Yondon M, Gray GC. Aerosol sampling in a hospital emergency room setting: a complementary surveillance method for the detection of respiratory viruses. Front Public Health 2018;6:174. 10.3389/fpubh.2018.00174 10.3389/fpubh.2018.00174 PMC6011129 29963543

[216] Rule AM, Apau O, Ahrenholz SH, Brueck SE, Lindsley WG, de Perio MA, et al. Healthcare personnel exposure in an emergency department during influenza season. PLoS ONE. 2018;13(8):e0203223. 30169507 10.1371/journal.pone.0203223 PMC6118374

[217] Yip L, Finn M, Granados A, Prost K, McGeer A, Gubbay JB, et al. Influenza virus RNA recovered from droplets and droplet nuclei emitted by adults in an acute care setting. J Occup Environ Hyg. 2019;16(5):341–8. 31050610 10.1080/15459624.2019.1591626 PMC7157967

[218] Mori T, Kikuchi T, Kato J, Koda Y, Sakurai M, Kikumi O, et al. Seasonal changes in indoor airborne fungal concentration in a hematology ward. J Infect Chemother. 2020;26(4):363–6. 31791593 10.1016/j.jiac.2019.10.020

[219] Mousavi MS, Hadei M, Majlesi M, Hopke PK, Yarahmadi M, Emam B, et al. Investigating the effect of several factors on concentrations of bioaerosols in a well-ventilated hospital environment. Environ Monit Assess. 2019;191(7):407. 31165312 10.1007/s10661-019-7559-0

[220] Shiu EYC, Huang W, Ye D, Xie Y, Mo J, Li Y, et al. Frequent recovery of influenza A but not influenza B virus RNA in aerosols in pediatric patient rooms. Indoor Air. 2020;30(5):805–15. 32201989 10.1111/ina.12669

[221] Stec J, Lenart-Boroń A. Assessment of microbiological aerosol concentration in selected healthcare facilities in southern Poland. Cent Eur J Public Health. 2019;27(3):239–44. 31580561 10.21101/cejph.a5681

[222] Zhao X, Nie W, Zhou C, Cheng M, Wang C, Liu Y, et al. Airborne transmission of influenza virus in a hospital of Qinhuangdao during 2017–2018 flu season. Food Environ Virol. 2019;11(4):427–39. 31549297 10.1007/s12560-019-09404-1

[223] Lane MA, Brownsword EA, Morgan JS, Babiker A, Vanairsdale SA, Lyon GM, et al. Bioaerosol sampling of a ventilated patient with COVID-19. Am J Infect Control. 2020;48(12):1540–2. 32763347 10.1016/j.ajic.2020.07.033 PMC7402277

[224] Lei H, Ye F, Liu X, Huang Z, Ling S, Jiang Z, et al. SARS-CoV-2 environmental contamination associated with persistently infected COVID-19 patients. Influenza Other Respir Viruses. 2020;14(6):688–99. 32578948 10.1111/irv.12783 PMC7361718

[225] Ge XY, Pu Y, Liao CH, Huang WF, Zeng Q, Zhou H, et al. Evaluation of the exposure risk of SARS-CoV-2 in different hospital environment. Sustain Cities Soc. 2020;1(61):102413. 10.1016/j.scs.2020.102413 PMC7375302 32834932

[226] Binder RA, Alarja NA, Robie ER, Kochek KE, Xiu L, Rocha-Melogno L, et al. Environmental and aerosolized severe acute respiratory syndrome coronavirus 2 among hospitalized coronavirus disease 2019 patients. J Infect Dis. 2020;222(11):1798–806. 32905595 10.1093/infdis/jiaa575 PMC7499634

[227] Guo ZD, Wang ZY, Zhang SF, Li X, Li L, Li C, et al. Aerosol and surface distribution of severe acute respiratory syndrome coronavirus 2 in hospital wards, Wuhan, China. Emerg Infect Dis [Internet]. 2020;26(7):1583–1591. 10.3201/eid2607.200885 10.3201/eid2607.200885 PMC7323510 32275497

[228] Jin T, Li J, Yang J, Li J, Hong F, Long H, et al. SARS-CoV-2 presented in the air of an intensive care unit (ICU). Sustain Cities Soc. 2021;1(65):102446. 10.1016/j.scs.2020.102446 PMC7428766 32837871

[229] Kenarkoohi A, Noorimotlagh Z, Falahi S, Amarloei A, Mirzaee SA, Pakzad I, et al. Hospital indoor air quality monitoring for the detection of SARS-CoV-2 (COVID-19) virus. Sci Total Environ. 2020;15(748):141324. 10.1016/j.scitotenv.2020.141324 PMC7387923 32805566

[230] Ma J, Qi X, Chen H, Li X, Zhang Z, Wang H, et al. Exhaled breath is a significant source of SARS-CoV-2 emission [Internet]. medRxiv; 2020 [cited 2023 Feb 2]. p. 2020.05.31.20115154. Available from: https://www.medrxiv.org/content/10.1101/2020.05.31.20115154v1

[231] Razzini K, Castrica M, Menchetti L, Maggi L, Negroni L, Orfeo NV, et al. SARS-CoV-2 RNA detection in the air and on surfaces in the COVID-19 ward of a hospital in Milan, Italy. Sci Total Environ. 2020;10(742):140540. 10.1016/j.scitotenv.2020.140540 PMC7319646 32619843

[232] Ding Z, Qian H, Xu B, Huang Y, Miao T, Yen HL, et al. Toilets dominate environmental detection of severe acute respiratory syndrome coronavirus 2 in a hospital. Sci Total Environ. 2021;20(753):141710. 10.1016/j.scitotenv.2020.141710 PMC7428758 32891988

[233] Jiang Y, Wang H, Chen Y, He J, Chen L, Liu Y, et al. Clinical data on hospital environmental hygiene monitoring and medical staff protection during the coronavirus disease 2019 outbreak [Internet]. medRxiv; 2020 [cited 2023 Feb 2]. p. 2020.02.25.20028043. Available from: https://www.medrxiv.org/content/10.1101/2020.02.25.20028043v2

[234] Lednicky JA, Lauzardo M, Fan ZH, Jutla A, Tilly TB, Gangwar M, et al. Viable SARS-CoV-2 in the air of a hospital room with COVID-19 patients [Internet]. medRxiv; 2020 [cited 2023 Feb 2]. p. 2020.08.03.20167395. Available from: https://www.medrxiv.org/content/10.1101/2020.08.03.20167395v1 10.1016/j.ijid.2020.09.025 PMC7493737 32949774

[235] Zhou J, Otter JA, Price JR, Cimpeanu C, Meno Garcia D, Kinross J, et al. Investigating severe acute respiratory syndrome coronavirus 2 (SARS-CoV-2) surface and air contamination in an acute healthcare setting during the peak of the coronavirus disease 2019 (COVID-19) pandemic in London. Clin Infect Dis. 2021;73(7):e1870–7. 32634826 10.1093/cid/ciaa905 PMC7454437

[236] Cabral CZ, Guaragna JB de A, Amantéa FC, Lopes PGM, Pasqualotto AC, Rhoden CR, et al. Distribution of airborne respiratory pathogens in pediatric emergency department waiting room. Pediatr Pulmonol. 2021;56(8):2724–8. 10.1002/ppul.25469 34185972

[237] Chamseddine A, Soudani N, Kanafani Z, Alameddine I, Dbaibo G, Zaraket H, et al. Detection of influenza virus in air samples of patient rooms. J Hosp Infect. 2021;1(108):33–42. 10.1016/j.jhin.2020.10.020 PMC7605760 33152397

[238] Tsay MD, Tseng CC, Wu NX, Lai CY. Size distribution and antibiotic-resistant characteristics of bacterial bioaerosol in intensive care unit before and during visits to patients. Environ Int. 2020;1(144):106024. 10.1016/j.envint.2020.106024 32795751

[239] Rufino de Sousa N, Steponaviciute L, Margerie L, Nissen K, Kjellin M, Reinius B, et al. Detection and isolation of airborne SARS-CoV-2 in a hospital setting. Indoor Air. 2022;32(3):e13023. 10.1111/ina.13023 PMC9111425 35347788

[240] Moore G, Rickard H, Stevenson D, Aranega-Bou P, Pitman J, Crook A, et al. Detection of SARS-CoV-2 within the healthcare environment: a multi-centre study conducted during the first wave of the COVID-19 outbreak in England. J Hosp Infect. 2021;1(108):189–96. 10.1016/j.jhin.2020.11.024 PMC7831847 33259882

[241] Ang AX, Luhung I, Ahidjo BA, Drautz-Moses DI, Tambyah PA, Mok CK, et al. Airborne SARS-CoV-2 surveillance in hospital environment using high-flowrate air samplers and its comparison to surface sampling. Indoor Air. 2022;32(1):e12930. 34519380 10.1111/ina.12930 PMC8653264

[242] Kotwa JD, Jamal AJ, Mbareche H, Yip L, Aftanas P, Barati S, et al. Surface and air contamination with severe acute respiratory syndrome coronavirus 2 from hospitalized coronavirus disease 2019 patients in Toronto, Canada, March–May 2020. J Infect Dis. 2022;225(5):768–76. 34850051 10.1093/infdis/jiab578 PMC8767887

[243] Ong SWX, Tan YK, Coleman KK, Tan BH, Leo YS, Wang DL, et al. Lack of viable severe acute respiratory coronavirus virus 2 (SARS-CoV-2) among PCR-positive air samples from hospital rooms and community isolation facilities. Infect Control Hosp Epidemiol. 2021;42(11):1327–32. 33487210 10.1017/ice.2021.8 PMC7870907

[244] Ghaffari HR, Farshidi H, Alipour V, Dindarloo K, Azad MH, Jamalidoust M, et al. Detection of SARS-CoV-2 in the indoor air of intensive care unit (ICU) for severe COVID-19 patients and its surroundings: considering the role of environmental conditions. Environ Sci Pollut Res. 2022;29(57):85612–8. 10.1007/s11356-021-16010-x PMC8418690 34482469

[245] Pochtovyi AA, Bacalin VV, Kuznetsova NA, Nikiforova MA, Shidlovskaya EV, Verdiev BI, et al. SARS-CoV-2 Aerosol and surface contamination in health care settings: the Moscow Pilot Study. Aerosol Air Qual Res. 2021;21(4):200604.

[246] López JH, Romo ÁS, Molina DC, Hernández GÁ, Cureño ÁBG, Acosta MA, et al. Detection of Sars-Cov-2 in the air of two hospitals in Hermosillo, Sonora, México, utilizing a low-cost environmental monitoring system. Int J Infect Dis. 2021;1(102):478–82. 10.1016/j.ijid.2020.10.089 PMC7607259 33157295

[247] Habibi N, Uddin S, Al-Salameen F, Al-Amad S, Kumar V, Al-Otaibi M, et al. SARS-CoV-2, other respiratory viruses and bacteria in aerosols: Report from Kuwait’s hospitals. Indoor Air. 2021;31(6):1815–25. 34121237 10.1111/ina.12871 PMC8447393

[248] Barbieri P, Zupin L, Licen S, Torboli V, Semeraro S, Cozzutto S, et al. Molecular detection of SARS-CoV-2 from indoor air samples in environmental monitoring needs adequate temporal coverage and infectivity assessment. Environ Res. 2021;1(198):111200. 10.1016/j.envres.2021.111200 PMC8065246 33901446

[249] de Man P, Paltansing S, Ong DSY, Vaessen N, van Nielen G, Koeleman JGM. Outbreak of coronavirus disease 2019 (COVID-19) in a nursing home associated with aerosol transmission as a result of inadequate ventilation. Clin Infect Dis. 2021;73(1):170–1. 32857130 10.1093/cid/ciaa1270 PMC7499506

[250] Mallach G, Kasloff SB, Kovesi T, Kumar A, Kulka R, Krishnan J, et al. Aerosol SARS-CoV-2 in hospitals and long-term care homes during the COVID-19 pandemic. PLoS ONE. 2021;16(9):e0258151. 34591919 10.1371/journal.pone.0258151 PMC8483369

[251] Zahedi A, Seif F, Golshan M, Khammar A, Rezaei Kahkha MR. Air surveillance for viral contamination with SARS-CoV-2 RNA at a healthcare facility. Food Environ Virol. 2022;14(4):374–83. 35610444 10.1007/s12560-022-09524-1 PMC9129059

[252] Azizi Jalilian F, Poormohammadi A, Teimoori A, Ansari N, Tarin Z, Ghorbani Shahna F, et al. Evaluation of SARS-CoV-2 in indoor air of Sina and Shahid Beheshti hospitals and patients’ houses. Food Environ Virol. 2022;14(2):190–8. 35212948 10.1007/s12560-022-09515-2 PMC8872858

[253] Izzotti A, Grasselli E, Barbaresi M, Bixio M, Colombo M, Pfeffer U, et al. Development of an integrated environmental monitoring protocol for SARS-CoV-2 contamination. Applications at the IRCSS San Martino Polyclinic Hospital in Genoa, Italy. Environ Res. 2022 Jun 1;209:112790. 10.1016/j.envres.2022.112790 PMC8800503 35104484

[254] Qiu G, Spillmann M, Tang J, Zhao YB, Tao Y, Zhang X, et al. On-site quantification and infection risk assessment of airborne SARS-CoV-2 virus via a nanoplasmonic bioaerosol sensing system in healthcare settings. Adv Sci. 2022;9(35):2204774. 10.1002/advs.202204774 PMC9762303 36310114

